# Biometrics-protected optical communication enabled by deep learning–enhanced triboelectric/photonic synergistic interface

**DOI:** 10.1126/sciadv.abl9874

**Published:** 2022-01-19

**Authors:** Bowei Dong, Zixuan Zhang, Qiongfeng Shi, Jingxuan Wei, Yiming Ma, Zian Xiao, Chengkuo Lee

**Affiliations:** 1 Department of Electrical and Computer Engineering, National University of Singapore, Singapore, Singapore 117583.; 2 Center for Intelligent Sensors and MEMS, National University of Singapore, Singapore, Singapore 117608.; 3 NUS Graduate School—Integrative Sciences and Engineering Programme (ISEP), National University of Singapore, Singapore, Singapore 119077.

## Abstract

Security is a prevailing concern in communication as conventional encryption methods are challenged by progressively powerful supercomputers. Here, we show that biometrics-protected optical communication can be constructed by synergizing triboelectric and nanophotonic technology. The synergy enables the loading of biometric information into the optical domain and the multiplexing of digital and biometric information at zero power consumption. The multiplexing process seals digital signals with a biometric envelope to avoid disrupting the original high-speed digital information and enhance the complexity of transmitted information. The system can perform demultiplexing, recover high-speed digital information, and implement deep learning to identify 15 users with around 95% accuracy, irrespective of biometric information data types (electrical, optical, or demultiplexed optical). Secure communication between users and the cloud is established after user identification for document exchange and smart home control. Through integrating triboelectric and photonics technology, our system provides a low-cost, easy-to-access, and ubiquitous solution for secure communication.

## Teaser

Biometrics-protected communication is realized by superimposing biometric signals on optical data using TENG/photonic interface.

## INTRODUCTION

Wearable flexible sensors have experienced vigorous development and advancement in the past decade because of their attractive characteristics such as flexibility, stretchability, light weight, and function diversity for widespread applications including personalized health care, soft robotics, prosthetics, and human-machine interfaces (HMIs) (*1*–*5*). In the era of the fifth generation (5G) mobile network and the Internet of Things (IoT), numerous sensors are expected to be connected wirelessly (*6*, *7*). These sensors serve as sensor nodes in a communication framework where the sensors and the cloud exchange data at an ultrafast data rate (*8*, *9*). Following this trend, a body area sensor network has been proposed to wirelessly connect wearable transmitters, receivers, and sensors with various functionalities to comprehensively monitor human health conditions (*10*, *11*). The traditional and dominant wearable sensors are based on the resistive mechanism and the capacitive mechanism (*12*, *13*). The need for external power supplies limits their widespread deployment in the IoT because of the power consumption issue and the battery replacement issue. The triboelectric nanogenerator (TENG), since its invention in 2012, has been explored as energy harvesters to drive wearable sensors due to its high output characteristics and wide adaptability (*14*–*18*). Benefiting from its versatile configurations and self-powered characteristics, TENG has been further investigated and deployed as self-powered sensors for motion monitoring and health care monitoring (*19*–*23*). TENG-based sensors have been integrated into diversified clothes to enable smart socks, belts, and wrist bands for gait analysis, driving status monitoring, and arterial pulse detection, respectively (*24*–*27*).

In addition to monitoring functions, TENG-based sensors can also provide promising control functions when configured into HMIs (*28*–*30*). Wearable HMIs are in burgeoning demand as an advanced solution to achieve human-machine interactions by virtue of their human state tracking capability (*31*, *32*). Versatile working mechanisms and structural configurations of TENGs have been used to implement different types of HMIs, such as touchpads, gloves, glasses, etc. (*33*–*35*). To enhance the control capability, various coding methods for TENG-based HMIs have been developed (*36*–*38*). Shi and Lee (*38*) developed a highly scalable and wearable control interface by encoding multidigit binary information into a spider net–shaped electrode layout, achieving a multidirectional three-dimensional (3D) control system with only one single electrode. Besides improved control capability, the TENG-based HMIs operate in a self-powered manner without the need for external power supplies, making them ideal sensor nodes in the communication framework of IoT systems. However, these HMIs face security issues in communication. Using current TENG-based HMIs, unauthorized users can also send commands and control designated entities. Potentially, TENG itself can address the security issue by incorporating the biometric identification function into HMIs (*39*, *40*). Recently, the use of TENG for biometric identification has been investigated. Deep learning (DL) analytics as a technique in artificial intelligence has been used to enhance the data analysis of TENG-based sensor signals to achieve biometric identification (*41*–*44*). Wu *et al*. (*41*) proposed a keystroke dynamics–based authentication system with TENG-based sensors to recognize the identity of users through their unique typing habits, which could potentially push cybersecurity to a new level without the concern of leaking passwords. Shi *et al.* (*42*) developed a smart floor monitoring system that can detect the walking position and gait-based identity of a user simultaneously by integrating DL analytics in TENG-based floor mats, enabling secure smart homes. Moving forward, it is desired to combine the biometric identification function and the control function into a single TENG-based sensor where control is only allowed after authority check, so as to enhance the system security.

To function as sensor nodes in IoT systems, TENG-based sensors should be able to transmit their signals to the cloud efficiently and robustly (*45*). Traditionally, signals from TENG-based sensors are captured by a microcontroller unit (MCU); then, the MCU controls a transmitter to send out the signals (*46*–*48*). This method involves many analog-to-digital and digital-to-analog conversions (ADC and DAC) and several communication interfaces, limiting the transmission efficiency and increasing the power consumption on the sensor end. To address this issue, a method based on electromagnetic coupling has been proposed (*49*, *50*). Two closed electrical circuits are placed in proximity, where one hosts the TENG-based sensor and the other hosts the receiver circuit. Upon human interaction, the current flow in the TENG circuit induces a current in the readout circuit via electromagnetic induction. Although the transmission efficiency is improved by removing the intermediate MCU, the transmission distance is limited because of the weak coupling between two circuits. Moreover, the electromagnetic coupling is vulnerable to electromagnetic interference (EMI), limiting their robustness for applications in complex IoT systems where strong EMI occurs because of the presence of numerous electrical components. In this regard, transmitting the TENG signals in the optical communication infrastructure could be advantageous. The optical communication is immune to EMI and can transmit ultrahigh-speed signals with low attenuation and low dispersion over a long distance. Nevertheless, it is challenging to load signals generated by TENG-based sensors into the optical domain efficiently and directly.

Combining triboelectric technology with nanophotonics technology could be a promising solution. Recently, the synergistic effect between triboelectric technology and aluminum nitride (AlN) photonics has been investigated (*51*, *52*). Two main advantages have been proven. On the one hand, thanks to the Pockels effect in AlN photonics (*53*, *54*), the coded control signals generated by TENG-based sensors in the form of high voltage can be loaded into the optical domain by the electro-optic effect without the need for external circuits or power supplies, resulting in optical strings of “ones” and “zeros” that carry the control information (*51*). On the other hand, when TENG-based sensors are used for monitoring functions, the sensory information can be recorded continuously and accurately because the capacitive nature of AlN photonic devices provides the open-circuit working condition for TENG (*52*). The availability of the open-circuit working condition can be attributed to the fact that the impedance of AlN photonic devices is several orders higher compared to TENG sensors because of their size difference. Benefiting from the two advantages, optical Morse code transmission and human-machine interaction in the virtual reality/augmented reality (VR/AR) space have been demonstrated using the triboelectric/photonics interface. However, the monitoring and transmission of biometric information have not been investigated using the triboelectric/photonics interface. Furthermore, the previous works did not incorporate the triboelectric/photonic interface into the optical communication infrastructure to exploit its main superior function of transmitting information at ultrahigh data rate. Whether the direct transmission of TENG-based sensor signals in the optical communication infrastructure will disrupt the digital information that is originally propagating in optical fibers remains a severe unknown issue that may hinder practical applications.

Here, we present a biometrics-protected optical communication technology as a low-cost, easy-to-access, and ubiquitous solution for secure communication between users (sensor nodes) and the cloud by leveraging on the DL-enhanced triboelectric/photonic synergistic interface. The synergistic interface is constructed on the basis of the fusion of flexible triboelectric biometric (TEB) scanner and AlN photonics-based biometric-optical information multiplexer (BOIMUX). The TEB scanner is a single-electrode TENG-based sensor that provides both the biometric identification function for secure communication and the control function for human-machine interactions. The system only enables the control function after the user authority is checked by biometric identification assisted with DL. Upon user interactions, enabled by the synergistic effect between triboelectric and nanophotonics, the interface loads biometric information into the optical domain and multiplexes biometric information and digital information that is originally propagating in optical fibers in a self-sustainable manner. The digital signals are sealed in a biometric envelope after multiplexing, thereby eliminating the communication latency and enhancing the complexity of transmitted information. The multiplexed signal in the form of a modulated wave packet is then transmitted efficiently and robustly to the cloud via the optical communication infrastructure. Because of the large frequency difference, low-frequency biometric information does not disrupt high-frequency digital information in the optical domain. In the cloud, the high-frequency digital information and low-frequency biometric information can be separated using fast Fourier transform (FFT) filters. Assisted with DL, biometric identification can be implemented to identify 15 users with around 95% accuracy and 23 users with around 90% accuracy irrespective of the data types of biometric information (electrical, optical, or demultiplexed optical), enabling secure communication between users and the cloud via the triboelectric/photonics interface. Secure exchange of high-speed documents and secure control of smart homes in the VR space are both demonstrated to prove the practicality of the proposed system.

## RESULTS

### Operation principle and architecture of biometrics-protected optical communication

**Fig. 1.**
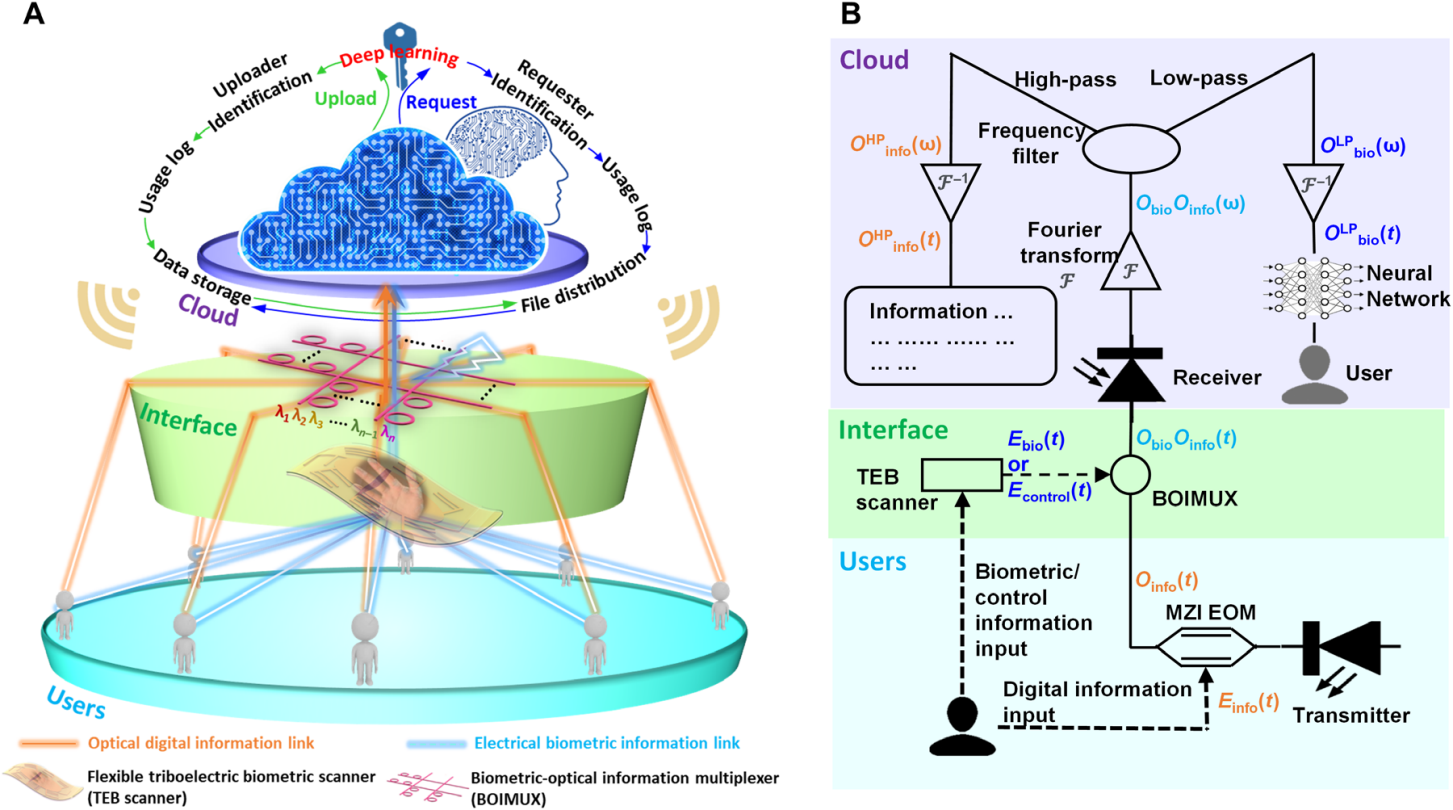
Architecture and operation principle of biometrics-protected optical communication. (**A**) Architecture with three components: users, interface, and cloud. (**B**) Detailed operation principle. LP, low-pass; HP, high-pass.

The system comprises three components: users, interface, and cloud (Fig. 1A). The users generate digital information, biometric information, and control information. The digital information is transmitted at high data rate in the optical communication infrastructure. The biometric and control information is generated when the user interacts with the interface. The interface features the combination of a TEB scanner and a BOIMUX, which synergistically loads the biometric information into the optical domain and multiplexes the digital information with biometric and control information. The cloud performs demultiplexing, then uses biometric information for user identification through DL, and decodes digital information. After the user identity is checked, the control authority is then granted to the user to control entities via the cloud.

The detailed operation principle in terms of information conversion and information flow is illustrated in Fig. 1B. Files to be uploaded are encoded as digital information *E*_info_ in the electrical domain. A Mach-Zehnder interferometer electro-optic modulator (MZI EOM) converts *E*_info_ into *O*_info_ in the optical domain as the optical input of BOIMUX. Meanwhile, users interact with the TEB scanner to generate biometric information *E*_bio_ in the electrical domain via the coupling effect of contact electrification and electrostatic induction. *E*_bio_ is applied to the BOIMUX. Because of the Pockels effect, *E*_bio_ modulates *O*_info_, resulting in *O*_bio_*O*_info_ as the output of BOIMUX. *O*_bio_ is the biometric information in the optical domain. Since the frequency of digital information is several orders of magnitude higher than biometric information, *O*_bio_*O*_info_ is a wave packet that simultaneously contains *O*_info_ as the signal and *O*_bio_ as the envelope. Therefore, *O*_info_ and *O*_bio_ can be separated in the frequency domain. In the cloud, *O*_bio_*O*_info_ is received. FFT filters are used to demultiplex *O*_bio_*O*_info_ into a high-frequency component *O*^HP^_info_ and a low-frequency component *O*^LP^_bio_. *O*^LP^_bio_ represents the biometric information and is propagated through a deep neural network (DNN) for user identification. After verifying the identity of users, *O*^HP^_info_ is decoded to ensure secure document transmission. Meanwhile, the control authority will be granted so that the users can use the same TEB scanner to generate control signals *E*_control_. *E*_control_ is then converted to *O*_control_ and similarly transmitted to the cloud as discussed above. Leveraging this system assisted by a single TENG-based sensor coupled to an AlN photonic device, further enhanced by DL analytics, document exchange and control can be implemented more securely.

### Co-design and characterization of TEB scanner and BOIMUX

**Fig. 2.**
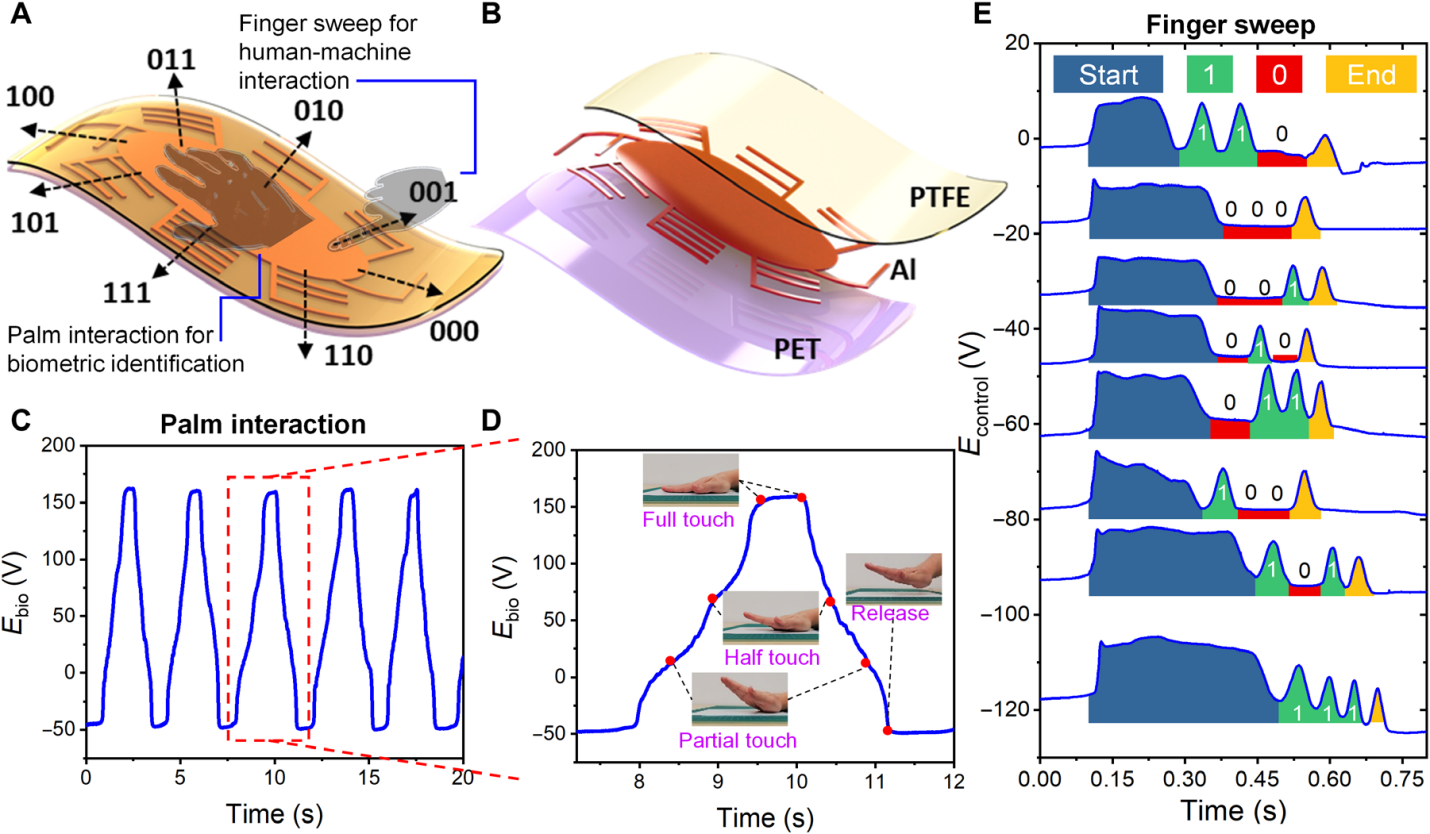
Design and characterization of TEB scanner. (**A**) Single-electrode TENG-based sensor with a central ellipse (contact separation–based single-electrode mode) for palm interaction to monitor biometric information and eight protruded digitated electrodes (sliding-based single-electrode mode) for finger sweep to record control information. (**B**) TEB scanner features an Al electrode sandwiched by a bottom polyethylene terephthalate (PET) layer for insulation and a top polytetrafluoroethylene (PTFE) layer as the triboelectric material, rendering low cost and ease of fabrication. (**C**) Self-generated *E*_bio_ spectrum under repeated human palm interaction. (**D**) Zoom-in of (C) to show the successful recording of palm characteristics with four characteristic states, namely, “partial touch,” “half touch,” “full touch,” and “release.” (**E**) Self-generated *E*_control_ spectrum when a finger sweeps across eight digitated electrodes. The resultant control signals are three-digit binary signals. “Start” corresponds to the moment when the finger touches the central ellipse and “End” corresponds to the moment when the finger sweeps across the outermost electrode bar.

To enable both the biometric identification function and the control function in a single TENG-based sensor and to load the corresponding signals into the optical domain efficiently and directly without disrupting the digital information in optical fibers, the triboelectric/photonic synergistic interface requires the co-design of TEB scanner and BOIMUX. The TEB scanner is a single-electrode TENG-based sensor that records both biometric information and control information (Fig. 2, A and B). The central ellipse works in contact separation–based single-electrode mode (fig. S1) for user palm monitoring. Eight digitated electrodes protrude from the central ellipse and work in sliding-based single-electrode mode (fig. S2) for control command recording. The optical image, power curve, and internal impedance of the manufactured TEB scanner are presented in figs. S3 and S4. The biometric information *E*_bio_ generated by palm interaction spans 200 V from −50 to 150 V (Fig. 2C). In an interaction cycle of 3 s, four characteristic states are identified, indicating that the TEB scanner can monitor palm characteristics (Fig. 2D). The control signal *E*_control_ generated by sweeping across eight digitated electrodes using a finger shows that the eight distinct three-digit binary codes can be distinguished (Fig. 2E). *E*_control_ spans approximately 10 V, which is an order lower than *E*_bio_. Although sweeping with more fingers at a faster speed can increase *E*_control_, *E*_control_ can hardly exceed 20 V (fig. S5).

The BOIMUX is an AlN microring resonator (MRR) EOM that performs electrical-to-optical (E-O) conversion on biometric and control information generated by the TEB scanner. The BOIMUX is designed to operate around 1550 nm, which is a standard working wavelength for optical communication with the lowest propagation loss. Because the TEB scanner adopts a minimalist design with only one shared electrode for cost optimization, the BOIMUX should preferably simultaneously have a high sensitivity to record control information of 20-V small voltages and a large sensing range to measure biometric information of 200-V large voltages.

Consequently, to cooperate with the TEB scanner, the BOIMUX meets three conditions that can be formulated as:

A) Nonzero transmission: For all *t* > 0, *O*_bio_(*t*) > 0 and *O*_control_(*t*) > 0. Otherwise, a small *O*_bio_ or *O*_control_ leads to *O*_bio_*O*_info_ ≈ 0 or *O*_control_*O*_info_ ≈ 0. Digital information is lost.

B) High sensitivity: $\text{There exists}\;\Delta V_{\text{control}}\leq 30\;V,\text{such that}\;|\int_{V_{\text{control}}}^{V_{\text{control}}+\Delta V_{\text{control}}}\frac{d(O_{\text{control}})}{d(V_{\text{control}})}dV_{\text{control}}|\geq 3\;\text{dB}$, so that BOIMUX has enough sensitivity to generate clear three-digit binary codes using 20-V small *E*_control_.

C) Large sensing range: $\text{For}\;\text{all}\;V_{\text{bio}}\in [-50\;V,150\;V],\frac{d(O_{\text{bio}})}{d(V_{\text{bio}})}\neq 0$. Otherwise, *O*_bio_ saturates. Biometric information is lost.

**Fig. 3.**
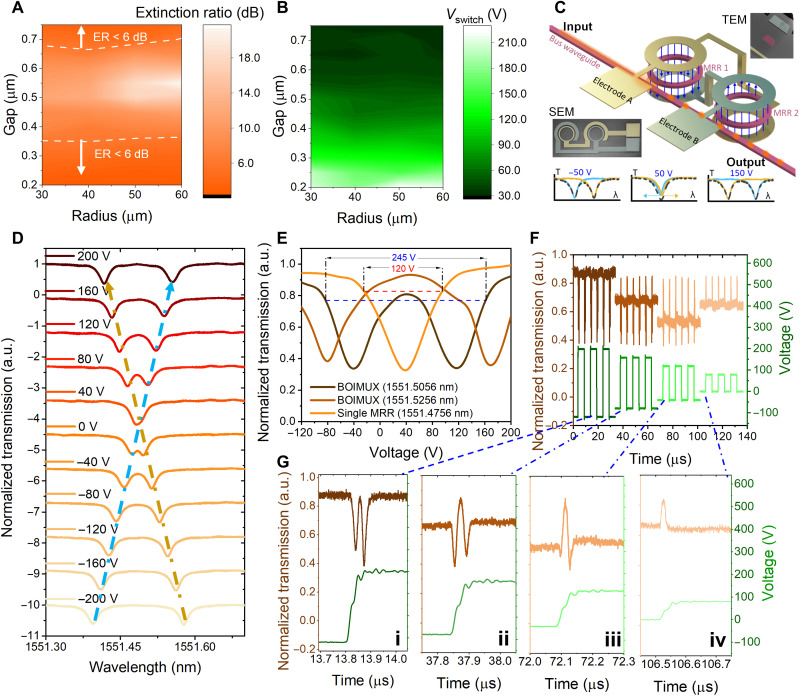
Design and characterization of BOIMUX. All presented transmissions are normalized. (**A**) ER map. ER ≤ 6 dB is required to secure nonzero optical transmission. (**B**) *V*_switch_ map. Small *V*_switch_ is required for high sensitivity to record the 20-V small *E*_control_. (**C**) CMRR design with push-pull electrode layout. Waveguides are sandwiched by the top and bottom electrodes (inset: TEM). The top electrode of MRR1 is connected to the bottom electrode of MRR2 and vice versa (inset: SEM). This design helps double the voltage sensing range without sacrificing sensitivity. (**D**) DC response of BOIMUX showing good linearity of resonant wavelength change with respect to applied voltages. a.u., arbitrary units. (**E**) Comparison of transmission-voltage curve in single MRR and BOIMUX. For a single MRR, only one trough is presented. The sensitive range is from −25 to 95 V. For BOIMUX, two troughs can be observed when the operation wavelength λ_op_ (1551.5056 or 1551.5256 nm) does not coincide with λ_mid_ (1551.4756 nm). The sensitive range is extended to cover −80 to 165 V at λ_op_ = 1551.5056 nm. (**F**) AC response of BOIMUX under different applied voltages. The working wavelength is 1551.5056 nm. (**G**) Zoom in of (F) for analyzing AC response in submicrosecond scale.

Condition (A) can be satisfied by engineering the radius *R* and coupling gap *g* of MRRs to ensure that the ratio between the highest optical transmission and the lowest optical transmission, i.e., extinction ratio (ER), is within 6 dB. Hence, *O*_bio_ and *O*_info_ are higher than 25% (equivalent to −6 dB), far exceeding 0. Arrays of MRRs are systematically studied to obtain the ER map with respect to *R* and *g*. A total of 48 MRRs that are formed by the combination of four different *R*’s (30, 40, 50, and 60 μm) and 12 different *g*’s (0.2 to 0.75 μm in the step of 0.05 μm) are investigated (fig. S6, A and B). The measured free spectral ranges are inversely proportional to *R* and do not depend on *g*. Figure S6 (C to F) shows all the spectra of the 48 MRRs, showing high fabrication uniformity and strong dependence of ER on *g*. The ER map shows that ER ≤ 6 dB is achieved in two regions: *g* ≤ 0.35 μm and *g* ≥ 0.7 μm (Fig. 3A). For condition (B), the switch voltage (*V*_switch_) is defined as the voltage required to realize Δ*O*_control_ = 3 dB. As shown in Fig. 3B, *V*_switch_ is negatively related to *g*. When *g* ≥ 0.7 μm, *V*_switch_ ≤ 30 V can be achieved. Hence, selecting *g* > 0.7 μm in a BOIMUX can satisfy both condition (A) and condition (B) at any examined *R*. However, the huge mismatch between *E*_bio_ and *E*_control_ makes it challenging to satisfy condition (B) and condition (C) simultaneously because the optical spectrum of any MRR features a Lorentzian line shape, whose sensitivity and sensing range are negatively related. A resonant line shape with 30-V *V*_switch_ and 6-dB ER only has a 120-V sensing range (as described in text S1 and fig. S7), which cannot cover −50 to 150 V. To tackle this challenge, the BOIMUX adopts a cascaded MRR (CMRR) design with a push-pull electrode layout (Fig. 3C). The sensing range can be doubled without compromising the sensitivity, as will be shown later. Therefore, using the CMRR design with push-pull electrode layout with *g* = 0.75 μm and *R* = 40 μm, the BOIMUX can satisfy all conditions (A), (B), and (C).

In the BOIMUX, the top electrode of MRR1 is connected to the bottom electrode of MRR2 and vice versa. Specifically, fig. S8 (A to C) shows false-colored scanning electron microscope (SEM) images of manufactured CMRRs under different magnifications, and fig. S8D shows a transmission electron microscope (TEM) image of the cross section labeled in fig. S8C. When *E*_bio_ or *E*_control_ is applied, opposite electric fields with the same amplitude penetrate through the two MRRs, leading to opposite resonant wavelength shifts (fig. S9). The DC response of BOIMUX shows good linearity of resonant wavelength change with respect to applied voltage (Fig. 3D), with a sensitivity of 0.4 pm/V (fig. S10). The BOIMUX doubles the sensitive voltage range from 120 to 245 V without sacrificing sensitivity (Fig. 3E). Note that the normalized optical transmission is symmetric with respect to around 50 V. It is designed on purpose to make most of the TEB scanner output (−50 to 150 V) fall in the sensitive range of the BOIMUX. The AC response of BOIMUX is studied by operating the BOIMUX at 1551.5056 nm and sequentially applying varying square waves (Fig. 3F). Figure 3G zooms in to Fig. 3F to analyze the dynamic response at submicrosecond scale. The observed AC response is well explained by the DC results, noting that Fig. 3G (i) is a replicate of the “1551.5056-nm” spectrum in Fig. 3E because the voltage rises linearly with respect to time. From Fig. 3G (ii) to (iii), the initial and final optical transmission decreases compared to Fig. 3G (i) because the starting voltage changes from −120 to −80 V and then to −40 V, as suggested in Fig. 3E. In Fig. 3G (iv), a single peak is observed because of the small voltage span that only covers the peak centering at 40 V in Fig. 3E. In addition, the distinct features shown in Fig. 3G imply that different *E*_bio_ carrying distinct user traits can be recorded. Using a 1-MHz square wave with 20-V *V*_pp_, the electrical information is accurately loaded into the optical domain (fig. S11). The high bandwidth advantageously guarantees that all palm interaction details can be recorded since the temporal resolution of human motion is at the kilohertz scale (*55*). It also proves that 20-V small voltages are measurable.

### Loading biometric and control information into optical domain without disrupting digital information

**Fig. 4.**
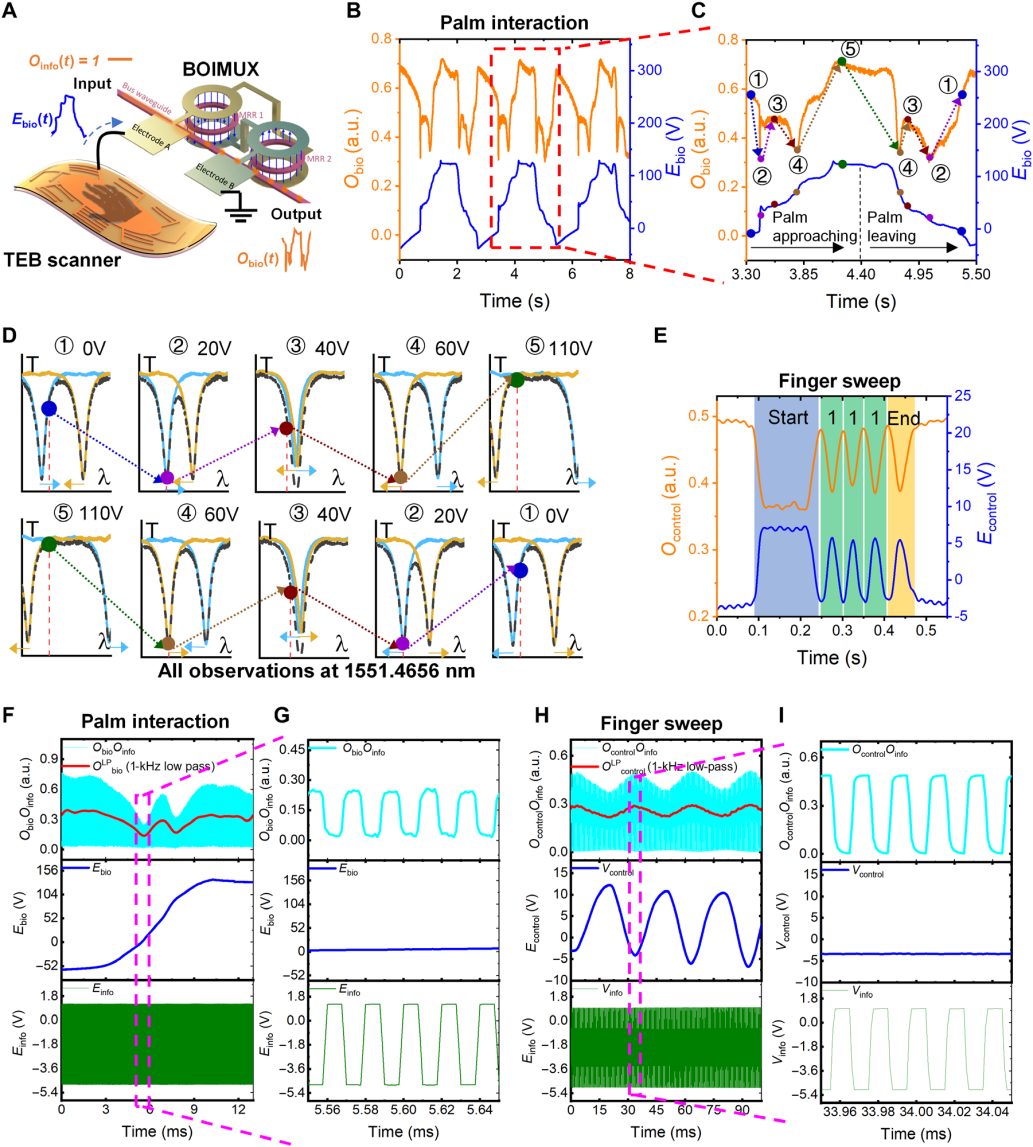
Loading biometric and control information into optical domain without disrupting digital information. The working wavelength is 1551.4656 nm. All presented transmissions are normalized. (**A**) Electrical connection between BOIMUX and TEB scanner. Electrode A of BOIMUX is connected to the single electrode of TEB scanner, while electrode B is grounded. (**B**) Conversion from *E*_bio_ to *O*_bio_ upon palm interaction. (**C**) Zoom in of (B) for the analysis of *E*_bio_ to *O*_bio_ conversion. (**D**) Explanation of the observed *E*_bio_ to *O*_bio_ conversion using schematic spectra of *O*_bio_ under different *E*_bio_’s at different states. (**E**) Conversion from *E*_control_ to *O*_control_ when finger sweeps across the 111 digitized electrode. (**F** to **I**) Multiplexed *O*_bio_*O*_info_ and *O*_control_*O*_info_ (cyan) generated by using *E*_bio_ and *E*_control_ (blue) to modulate *O*_info_ (green) and the corresponding extracted biometric information *O*^LP^_bio_ and control information *O*^LP^_control_ (red) using a 1-kHz FFT low-pass filter. (F) Upon palm interaction. (G) Zoom in of (F). (H) Upon finger sweep. (I) Zoom in of (H).

The primary function of the triboelectric/photonic interface is to record and perform E-O conversion on biometric and control information. Therefore, the top electrode of MRR1 of BOIMUX is connected to the single electrode of TEB scanner, while the top electrode of MRR2 is grounded (Fig. 4A). The biometric information *E*_bio_ and the corresponding converted *O*_bio_ of three consecutive palm interaction cycles are plotted in Fig. 4B. *E*_bio_ keeps the features shown in Fig. 1D. Meanwhile, *O*_bio_ shows a dynamic response similar to Fig. 3G (i), indicating that the triboelectric/photonic interface maintains the performance of TEB scanner and BOIMUX in the E-O conversion process. The analysis of one interaction cycle (Fig. 4C) shows that the E-O conversion process can be explained by the push-pull mechanism (Fig. 4D). When the palm approaches the TEB scanner, *E*_bio_ increases and *O*_bio_ undergoes five characteristic states (① to ⑤). When the palm leaves the TEB scanner, *E*_bio_ decreases and the five characteristic states are experienced in the reversed order (⑤ to ①). At state ①, *E*_bio_ is around 0 V, and *O*_bio_ is around 0.6. From state ① to state ②, *E*_bio_ rises from 0 V to around 20 V, λ_1_ (blue spectrum) redshifts to 1551.4656 nm, resulting in the lowest *O*_bio_ of 0.3. As *E*_bio_ further increases from 20 to 40 V during state ② to state ③, λ_1_ and λ_2_ (yellow spectrum) coincide at 1551.4756 nm, leading to a moderate *O*_bio_ of 0.5 at 1551.4656 nm. From state ③ to state ④, *E*_bio_ increases from 40 to 60 V, λ_2_ blueshifts to 1551.4656 nm, causing the lowest *O*_bio_ again. As *E*_bio_ increases to more than 100 V at state ⑤, λ_1_ and λ_2_ are driven to wavelengths that are far away from 1551.4656 nm, resulting in the highest *O*_bio_ of more than 0.7. After state ⑤, *E*_bio_ gradually falls from more than 100 to −30 V, and the corresponding *O*_bio_ spectra follow the reverse process of state ① to state ⑤ and will not be discussed in detail here. Next, the loading to control information *E*_control_ generated by finger sweep into the optical domain as *O*_control_ is examined. Figure 4E demonstrates the conversion and recognition of the three-digit binary code “111.” The corresponding E-O conversion process is explained in fig. S12. The other seven three-digit binary codes can also be successfully converted and recognized (fig. S13), confirming the general control capability of the interface. In the current BOIMUX, the small Pockels coefficient of AlN limits the sensitivity. Only large biometric signals generated by strenuous human motion can be recorded. Other photonic materials with larger Pockels coefficients such as lithium niobate (LiNbO_3_) or barium titanate (BaTiO_3_) (*53*) can be used for subtle human motion recording (such as voice or eye motion) (*35*, *56*). The required footprint of TEB scanner can be reduced accordingly.

While the biometric and control information is efficiently and directly loaded into the optical domain, it should not disrupt the digital information that is originally propagating in the optical fibers. Otherwise, the high-speed data transmission advantage of optical communication will be inhibited. Through the proper co-design, the triboelectric/photonic interface is able to multiplex digital information with biometric and control information while loading. Figure 4 (F and G) shows the individual digital information and biometric information in the electrical domain (*E*_info_, bottom green; *E*_bio_, middle blue) and the multiplexed signal in the optical domain (*O*_bio_*O*_info_, top cyan). The upper envelope of *O*_bio_*O*_info_ has similar characteristics as *O*_bio_ in Fig. 4A, suggesting that *O*_bio_*O*_info_ effectively carries the biometric information. The biometric information can be extracted using an FFT low-pass filter to obtain the resultant *O*^LP^_bio_ (red curve). Digital information is well preserved in *O*_bio_*O*_info_ (Fig. 4G top), suggesting the successful multiplexing of digital information and biometric information. Besides simple digital information, the multiplexing of modulated digital information and biometric information is also studied. In fig. S14, the original digital information is amplitude-modulated, frequency-modulated, and phase-modulated, respectively. In all three cases, the biometric information can be extracted as *O*^LP^_bio_ using FFT low-pass filters. Furthermore, the original digital information is not disturbed. In the case of control information generated by finger sweep, the multiplexing is still effective (Fig. 4, G and H, and fig. S15).

### DL-enhanced biometrics-protected optical communication and its applications

**Fig. 5.**
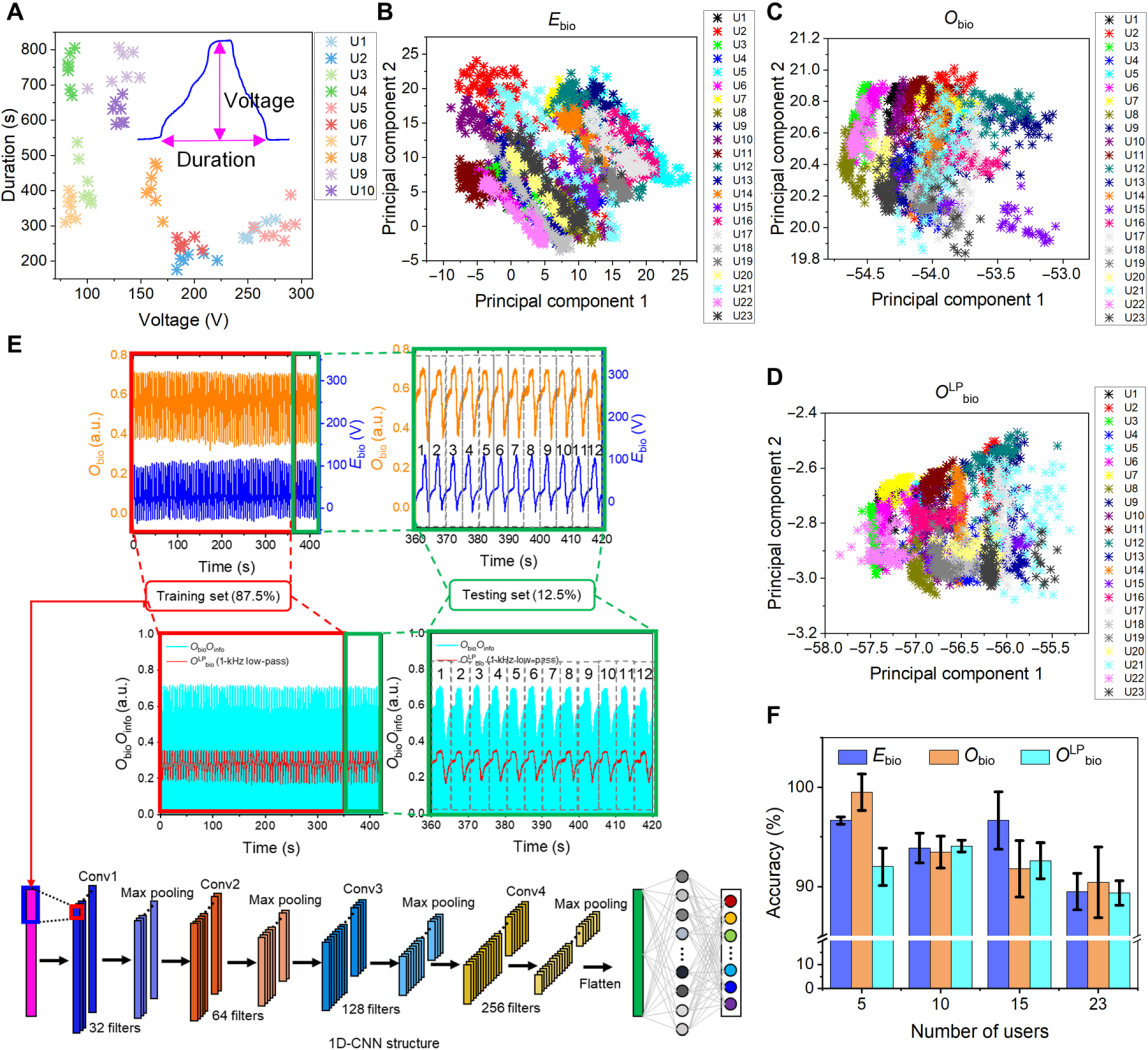
DL-enabled biometric identification rationality and results. All presented transmissions are normalized. (**A**) Distinguish 10 users using the voltage span and duration of *E*_bio_. Each user contains seven samples. (**B** to **D**) 2D-PCA results of biometric information in the form of (B) *E*_bio_, (C) *O*_bio_, and (D) *O*^LP^_bio_. Eighty-four samples are collected from each of the 23 users. Each sample contains 1600 temporal data points. Thus, 1600 by 1932 matrices are used for 2D-PCA. (**E**) Dataset division and detailed 1D-CNN structure. A total of 96 × 23 samples are divided into two portions, 84 × 23 (87.5%) as the training set and 12 × 23 (12.5%) as the testing set. (**F**) Accuracy of using DL-enabled biometric identification for three different biometric information data types (*E*_bio_, *O*_bio_, and *O*^LP^_bio_) when 5, 10, 15, or 23 users are involved.

To enhance security, the system requires the users to upload their biometric information for identification before permitting the control authority. The biometric information is recorded by the TEB scanner upon user palm interaction. In principle, the recorded biometric information is high-dimensional information with embedded user traits. Therefore, using DL based on convolutional neural networks (CNNs) might help with feature extraction and user identification. However, before implementing DL, we evaluate the existence of user traits in the recorded behavioral biometric information. To find physical evidence, the two most prominent features of *E*_bio_ in one user palm interaction cycle are analyzed, i.e., voltage span and duration (Fig. 5A, inset). Using the voltage-duration map, 10 users can be approximately distinguished (Fig. 5A). However, strong confusion happens when 23 users are considered (fig. S16). 2D principal components analysis (2D-PCA) is then used to find statistical evidence. To obtain a data size big enough for statistical analysis using 2D-PCA and the subsequent DL-enabled biometric identification, 96 samples are collected from each of the 23 users (labeled as user 1 to user 23). Each sample has 1600 temporal data points. One typical sample of different data types (electrical, optical, or demultiplexed optical) generated by each user is presented in figs. S17 to S19. *E*_bio_, *O*_bio_, and *O*_bio_*O*_info_ are all recorded for comparison. The recorded spectra illustrate that the conversion and the use of FFT low-pass filters do not deteriorate the data quality. For 2D-PCA, 84 samples of the 96 samples from each user are used. Thus, the matrix is a 1600 by 1932 matrix (fig. S20). The distribution of 1932 samples (84 from each of 23 users) shows that 23 users can be approximately distinguished in the PC1-PC2 space (Fig. 5B). It provides a stronger piece of evidence to support the existence of user traits that justifies the use of DL-enabled analytics. Since the system involves E-O conversions, the multiplexing of *O*_info_ and *O*_bio_, and the FFT filtering of *O*_bio_*O*_info_, the data robustness is important to ensure that DL-enabled biometric identification can maintain a high accuracy if practically the FFT-filtered biometric information *O*^LP^_bio_ is used. Figure 5C proves that the 2D-PCA classification can still be done if *O*_bio_ is used. Even *O*^LP^_bio_ can be distinguished (Fig. 5D). The cumulative variance plot shows that the first 20 PCs in *E*_bio_ and *O*_bio_ can account for 99% total variance, while the first 10 PCs in *O*^LP^_bio_ can account for the same variance (fig. S21). A 1D-CNN is used to implement DL. The dataset division and detailed 1D-CNN structure are shown in Fig. 5E. A total of 96 × 23 samples are divided into two portions, 84 × 23 (87.5%) as the training set, and 12 × 23 (12.5%) as the testing set. The effect of different numbers of users and different data formats (*E*_bio_, *O*_bio_, and *O*^LP^_bio_) on identification accuracy is investigated by comparing 12 cases (four numbers of users and three data formats). To obtain reliable results for comparison, the DL analysis is implemented 20 times for each case. The mean accuracies are reported as columns in Fig. 5F with the SD as error bars. The three types of biometric information present similar accuracy when 5, 10, 15, or 23 users are involved, and all maintain around 90% accuracy in identifying 23 users. Typical confusion maps of all scenarios are presented in figs. S22 to S29. The implementation of DL-enabled biometric identification is thus successfully demonstrated. Besides directly using the instantaneous time-varying finite signal as the input to implement DL, wavelet transform–enhanced DL is also investigated (text S2 and figs. S30 to S32). The wavelet transform helps convert the original instantaneous time-varying finite signal into a 2D map that contains both the frequency and time information. The 2D map is then used as the input for a 2D-CNN. The resultant identification accuracy using the wavelet transform–enhanced DL method is comparable to that using the original instantaneous time-varying finite signal. The comparable accuracy could be possibly attributed to the lack of frequency information in the biometric signal. The frequencies of all the biometric signals are very low and thus do not provide substantial features to classify different users.

**Fig. 6.**
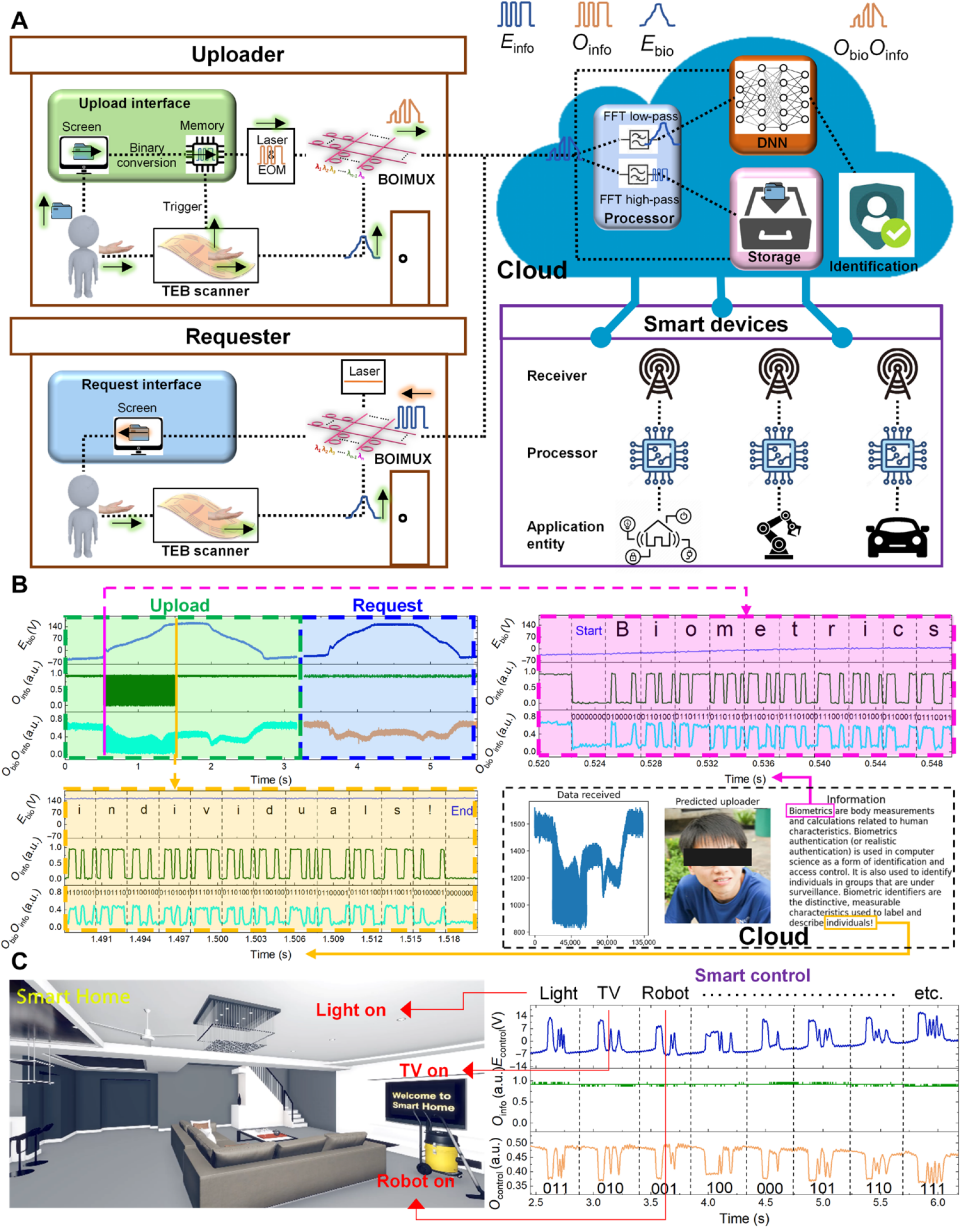
Demonstration of applications using biometrics-protected optical communication. (**A**) Operation principle of demonstrations. Uploaders use an upload interface, TEB scanner, and BOIMUX to upload files to the cloud. The cloud implements data demultiplexing, identification, display, and storage. Requesters use TEB scanner and BOIMUX to send their biometric information for identification. Upon successful request, the requester will receive the requested file (in document exchange) or be able to control smart devices via the cloud (in smart home control). (**B**) Exemplary upload and request waveforms and the receiving interface. User identification and authority check are successful. The text file in the form of digital information is embedded in the multiplexed waveform *O*_bio_*O*_info_ (cyan). The information is correctly decoded and displayed as a paragraph starting with Biometrics (pink) and ending with individuals! (yellow). The difference between upload and request from the same user is the existence of meaningful *O*_info_. During upload, *O*_info_ contains digital information that can be effectively carried over by *O*_bio_*O*_info_ and captured and recovered by the cloud. In contrast, *O*_info_ will be constantly 1 in the request process. (**C**) Exemplary control waveforms and the controlled smart home in the VR space. The three-digit binary codes 011, 010, and 001 are used for light, TV, and sweeping robot control, respectively.

To present system practicality, we demonstrate biometrics-protected document exchange and smart home control in the VR space. As shown in Fig. 6A, in the file upload process, the uploader inputs via the uploader interface. The file is converted to digital information *E*_info_ and stored in memory. To ensure that the digital information and biometric information are generated simultaneously so that they can be multiplexed, another TENG device (called trigger TENG) with a structure similar to the TEB scanner is placed beneath the TEB scanner (fig. S33). The TEB scanner is connected to the BOIMUX, while the trigger TENG is connected to an MCU that controls the laser and EOM. When the user interacts with the TEB scanner, biometric information *E*_bio_ is generated. Meanwhile, the TEB scanner presses the trigger TENG and leads to a pulse trigger signal that activates the generation of digital information *O*_info_. *E*_bio_ modulates *O*_info_ at BOIMUX and generates *O*_bio_*O*_info_. *O*_bio_*O*_info_ arrives at the cloud and is filtered as *O*^LP^_bio_ using an FFT low-pass filter. *O*^LP^_bio_ is then used for DNN inference. Upon successful authority identification, the digital information is extracted from *O*_bio_*O*_info_ using an FFT high-pass filter and decoded. In the file request process, the requester delivers the biometric information to the cloud similar to the upload process, but no digital information uploading is involved. The detailed upload and request mechanism and real-life demonstration of upload and request are shown in movies S1 to S4. An exemplary upload and request waveform, together with the receiving interface at the cloud, are highlighted in Fig. 6B. As shown in the pink and the yellow box corresponding to the start and the end of digital information, respectively, the two words “Biometrics” and “individuals!” are successfully transmitted and displayed. The data transmission speed of the current system is limited by the bandwidth of the MCU. However, using faster data acquisition systems or even potential all-optical ADC, the high-speed advantage of optical transmission can be fully exploited. For instance, the current bit rate of optical information can exceed 400 Gbps using wavelength-division multiplexing (*57*).

Besides biometrics-protected document exchange, the system also allows biometrics-protected smart home control in the VR space. Upon successful authority identification, the user can send control signals *E*_control_ to the cloud using the TEB scanner control interface. Different control commands are defined by different digitated electrodes. As shown in Fig. 6C, the light, TV, and sweeping robot can be turned on by control signals “011,” “010,” and “001,” respectively. The real-life demonstration of smart home control in the VR space is shown in movie S5.

Traditionally, encryption algorithms were adopted for optical communication security. Although physiological biometrics based on human images were recently used to improve security, there are still privacy and cost issues. Our demonstrations show that behavioral biometrics assisted with DL can enhance optical communication security while maintaining good privacy and cost-effectiveness. Digital information and biometric information can be multiplexed by leveraging the triboelectric/photonic synergistic interface, suggesting that the deployment of biometrics-protected optical communication can be built on the existing high-speed optical communication infrastructure to achieve value-added.

## DISCUSSION

Biometrics-protected optical communication is realized by a system using the DL-enhanced triboelectric/photonic synergistic interface that provides biometric-traits/control-commands superimposed digital data in the high-speed optical communication infrastructure for secure data access and transmission in the cloud. Our system offers many features including the recording of both biometric information and control information using a single TENG-based sensor with low-cost and minimized power consumption, the ability to load biometric and control information generated by TENG-based sensors into the optical domain without disrupting the original digital information propagating in optical fibers, and the high identification accuracy for various types of biometric information using DL. First, the TEB scanner in the form of a single TENG-based sensor is designed with a central ellipse for biometric information monitoring and surrounding protruded digitate electrodes for control information recording. The signals are generated on the basis of contact electrification through user-scanner interaction, which effectively converts human motion into electricity in a cost-effective manner. Second, the synergy between the triboelectric effect and the Pockels effect further enables the direct loading of biometric and control information into the optical domain without the need for external circuits or power supply, minimizing cost and power consumption. By leveraging the large frequency difference between digital information and biometric and control information, multiplexing can be realized via the co-design of TEB scanner and BOIMUX to avoid disrupting digital information. After multiplexing, digital signals are sealed with a biometric envelope to enhance the complexity of transmitted information. Third, DL is used to analyze the complex and high-dimensional biometric information, resulting in ~95% accuracy for biometric identification of 15 users and ~90% for 23 users. The accuracy does not decay when biometric information is in the form of original electrical signals, converted optical signals, and even demultiplexed optical signals. Although alternative technologies such as physical unclonable functions and quantum key distribution are theoretically unbreakable and potentially provide stronger security, they require complex, high-end, and expensive hardware support, impeding user-friendly and low-cost applications. In contrast, our proposed system offers a solution for low-cost and easy-to-access optical communication architecture with a great degree of security as a potential ubiquitous solution. Its potential to integrate numerous sensors via various optical multiplexing methods for DL-based multisensor data fusion (*58*) and to directly interface accelerated photonic neural networks (*59*, *60*) for efficient edge computing could lead to more secure and accurate systems with stronger computation power for communication in the DL-enabled 5G and IoT era.

## MATERIALS AND METHODS

### Fabrication of the TEB scanner

The TEB scanner was fabricated by a low-cost and facile process. First, an unmodified polytetrafluoroethylene (PTFE) thin film of 100-μm thickness was cut into an ellipse shape. Next, Al tapes as the sensing electrode were cut into thin bars and an ellipse shape but with a smaller size than the PTFE thin film to match common human fingers and palms. The obtained Al components were then attached to the backside of the PTFE thin film, with the ellipse-shaped electrode in the middle and the thin-bar electrodes at the surroundings according to the specifically designed coding patterns. All the Al electrodes are connected, forming the single-electrode interface for both palm and finger interactions. Then, a thin copper wire was connected to the Al electrode to collect the generated output signals during various human interactions. After that, a whole layer of double-sided adhesive tape was applied to the previously fabricated Al/PTFE part. Last, a polyethylene terephthalate (PET) thin film (thickness, 100 μm) with the same size as the PTFE layer was attached and served as the insulation/protection layer and the supporting substrate for the TEB scanner. Hence, the final configuration was PET/Al/PTFE, from bottom to top.

### Characterization of the TEB scanner

A programmable electrometer (Keithley, 6514) was used to test the open-circuit voltage, and an oscilloscope (Agilent, DSO-X3034A) was connected to the electrometer using a BNC cable for real-time data acquisition. The positive electrode of the electrometer was connected to the single electrode of the TEB scanner. The negative electrode of the electrometer was connected to the ground.

### Fabrication of BOIMUX

BOIMUX was fabricated by a standard complementary metal-oxide semiconductor manufacturing process. The fabrication started from 8-inch SiO_2_-covered Si wafers. A 120-nm TiN layer and 50-nm Si_3_N_4_ layer were deposited and patterned as bottom electrodes. A 2-μm SiO_2_ was deposited and planarized as waveguide bottom cladding. Next, a 400-nm AlN layer was deposited followed by the deposition of a 200-nm SiO_2_ layer as the hard mask. The waveguide patterns were defined using deep ultraviolet lithography and then etched using deep reactive-ion etching. After waveguide formation, another 2-μm SiO_2_ was deposited and planarized as waveguide top cladding. Contact holes were etched for the bottom TiN electrodes. Last, a 2-μm Al layer was deposited and patterned as top electrodes and contacts to bottom TiN electrodes.

### Characterization of BOIMUX

#### Characterization of passive performance

A tunable laser (Keysight, 81960A) was used as the light source. The light entered a single mode–maintaining polarization controller for polarization control. Then, the light was focused into the on-chip AlN waveguide using a tapered fiber (OZ Optics, TSMJ-3A-1550-9/125-0.25-18-2.5-14-3-AR). After on-chip routing, the light was collected by another tapered fiber and directed to a power sensor (Keysight, 81636B). The tunable laser and the power sensor formed an integrated system provided by Keysight. The wavelength scanning was controlled using a commercial software offered by Keysight as well (Keysight; IL Engine, Photonic Application Package Manager, N7700A Photonic Application Suite). The computer to laser/power sensor integrated system connection was enabled by a USB/GPIB adapter (Keysight, 82357B)

#### Characterization of active performance

For DC performance characterization, the optical setup was the same as in the “Characterization of passive performance” section. A tunable DC voltage supply (Agilent, E3631A) was used. The voltage was amplified by 20 times using a voltage amplifier (FLC Electronics, A400DI) and then applied to BOIMUX via a ground-signal-ground (GSG) probe (MPI, T26A GSG100). For AC performance characterization, a waveform generator (HP, 33120A) was used instead of a DC voltage supply. The rest of the electrical setup was the same as DC performance characterization. In the optical setup, since Keysight 81636B is not compatible with high-speed measurement, a high-speed photodetector (Thorlabs, DET08CFC/M) was used instead. An erbium-doped fiber amplifier was applied before the high-speed photodetector so that the photodetector output was high enough to be captured by an oscilloscope (Agilent, DSO-X3034A) for dynamics analysis. The rest of the optical setup was the same as DC performance characterization.

### Converting biometric information from the electrical domain to the optical domain

The working wavelength was 1551.4656 nm. The whole setup was generally the same as in the “Characterization of active performance” section, but neither the DC voltage supply nor the waveform generator together with the voltage amplifier was required. Electrode A of BOIMUX was connected to the single electrode of the TEB scanner, while electrode B was grounded. The TEB scanner directly applied voltage to BOIMUX without the need for external circuits or power supplies.

### Multiplexing digital information and biometric information

The working wavelength was 1551.4656 nm. The whole setup was generally the same as in the “Converting biometric information from the electrical domain to the optical domain” section. However, the light entered a commercial EOM (Thorlabs, LN81S-FC) directly after the light was emitted from the tunable laser. A waveform generator (HP, 33120A) was connected to the EOM for converting digital information from the electrical domain into the optical domain. After the modulated light that carried the digital information passed through a polarization controller and entered BOIMUX, it was further modulated by the voltage from the TEB scanner that carried the biometric information. Consequently, the output light from BOIMUX carried both the digital information as a high-frequency signal and the biometric information as a low-frequency envelope.

### Data collection and DL training model

#### Data collection and dataset

In the data collection process, the participants were asked to interact with the TEB scanner using their palms in a predefined manner (i.e., in the order of partial touch, half touch, full touch, and release, as shown in Fig. 2D). This predefined manner helps collect as much temporal palm interaction information as possible. The sample data were collected by the same method as in the “Multiplexing digital information and biometric information” section. However, the real-time sample data from the photodetector were first sent to an amplifying circuit, then filtered by a low-pass filter to reduce noise, and lastly acquired by the signal acquisition module in a MCU (STM32F746ZG) embedded in a development board (STMicroelectronics, NUCLEO-F746ZG). The acquired signals were sent to a laptop via USB cable communication with a baud rate of 1.3824 MHz/s limited by the MCU. Each sample spanned 5 s and was smoothened to 1600 data points. The data collection process involved 23 users, labeled as user 1 to user 23. For each user, 96 samples were collected, leading to a total of 2208 samples. A total of 1932 samples (87.5%) were used as the training set, while the other 276 samples (12.5%) were used as the testing set.

#### DL training model

A 1D-CNN was trained on a standard consumer-grade computer to predict the 23 different users, given biometric information (palm interaction) of each person. The 1D-CNN was developed in Python with a Keras and TensorFlow backend. The network consisted of four convolution layers, each of which was followed by a MaxPooling1D layer. These layers extracted patterns from the time-sequent samples, and the resulting embedding was then passed through a fully connected layer to lastly output the predicted user. The network was trained using stochastic gradient descent to minimize the mean squared error between the predicted user and the labeled user. More specifically, we trained the network with a batch size of 50 and with a learning rate of 0.0001 using the adaptive moment estimation (Adam) optimizer. After training the 1D-CNN for 500 epochs, the inference process was implemented for the testing set.

### Demonstration of applications using biometrics-protected optical communication system

#### Document exchange (upload)

On the uploader side, the text document was converted to digital information following the American Standard Code for Information Interchange (ASCII), where each character contains 8 bits. The whole setup was generally the same as in the “Multiplexing digital information and biometric information” section. However, instead of a waveform generator, the commercial EOM was controlled by an MCU (Arduino, MEGA 2560). The Arduino generated meaningful digital information with a bit rate of 300 μs/bit. Hence, the Arduino could transmit 1/(300 × 10^-6 × 8) ≈ 417 characters per second.

On the cloud side, a development broad (STMicroelectronics, NUCLEO-F746ZG) was used to collect the analog signal from the amplifying circuit after photodetector and convert the analog signal to digital signal using the built-in ADC. The development board was connected to a PC via USB cable communication to transmit the voltage information (VI) with a rate of 37.5 μs per VI, and every eight VIs represented one bit at the receiver side for stable and reliable transmission. Therefore, at the receiver side, 1 s/37.5 μs/8 = 3333.3 bits/s was received, corresponding to 3333.3/8 ≈ 417 characters per second (same as uploader side). Then, the PC started to process the data. First, the acquired signals spanning 5 s went through an FFT low-pass filter (with a sampling frequency of 1 kHz and cut-off frequency of 20 Hz) developed in Python environment to extract the biometric information *O*^LP^_bio_. *O*^LP^_bio_ was smoothened to 1600 data points and then recognized by the trained DL model to predict the user. If the authority check failed, the document exchange process terminated immediately. If the authority check succeeded, the acquired signals went through an FFT high-pass filter and then divided into high values (as “1’s”) and low values (as “0’s”) by a threshold to convert the signals into binary data. The binary data were separated by 8-bit intervals and decoded back to the original text information using ASCII. The text information was lastly displayed and stored in the cloud.

#### Document exchange (request)

The difference between upload and request from the same user was the existence of meaningful *O*_info_. During upload, *O*_info_ contained digital information that could be carried over by *O*_bio_*O*_info_ and then captured and recovered by the cloud. In contrast, *O*_info_ will be constantly “1” in the request process.

#### Smart home control in the VR space

The whole setup was generally the same as in the “Document exchange (upload)” section. A piece of biometric information was firstly sent to the cloud to request. The request process was similar to the “Document exchange (request)” section. If the request succeeded, i.e., the DL model prediction indicated control authority, control commands generated by the TEB scanner control interface would be sent to Unity 3D interface in the cloud to enable smart home control.

All the performed experiments in this work complied with a protocol approved by the National University of Singapore Institutional Review Board. All participation subjects were volunteers, and informed consent was obtained before participation in the experiments.

## Notes

View/request a protocol for this paper from *Bio-protocol*.
