# Supplementary material for: Biometrics-protected optical communication enabled by deep learning–enhanced triboelectric/photonic synergistic interface
Source: Sci Adv. 2022 Jan 19;8(3):eabl9874. doi: 10.1126/sciadv.abl9874 (PMC8769542; doi:10.1126/sciadv.abl9874)
Supplement: Supplementary file 1 — Text S1 and S2 Figs. S1 to S33 Legends for movies S1 to S5 [file sciadv.abl9874_sm.pdf]

Science Advances
AAAS

Supplementary Materials for

Biometrics-protected optical communication enabled by deep learning–enhanced triboelectric/photonic synergistic interface

Bowei Dong, Zixuan Zhang, Qiongfeng Shi, Jingxuan Wei, Yiming Ma, Zian Xiao, Chengkuo Lee*

*Corresponding author. Email: elelc@nus.edu.sg

Published 19 January 2022, *Sci. Adv.* **8**, eabl9874 (2022)
DOI: 10.1126/sciadv.abl9874

The PDF file includes:

Text S1 and S2
Figs. S1 to S33
Legends for movies S1 to S5

Other Supplementary Material for this manuscript includes the following:

Movies S1 to S5

Supplementary Text S1

Analysis of sensing range using single MRR

According to the coupled-mode theory (CMT), the transmission from a single MRR is expressed as:

$$P_t = \frac{\alpha^2+|t|^2-2\alpha|t|\cos(\theta+\varphi_t)}{1+\alpha^2|t|^2-2\alpha|t|\cos(\theta+\varphi_t)} \qquad \textbf{Equation S1}$$

where α is the loss coefficient of the ring, t is a coupling constant, θ is phase introduced by a round trip in the MRR, φ_t is the phase introduced by coupling.

P_t is related to wavelength λ by:

$$\theta = 4\pi^2 n_{eff} \frac{R}{\lambda} \qquad \textbf{Equation S2}$$

where n_{eff} is the effective refractive index of the propagating mode, R is the MRR radius, which is 40 μm in the BOIMUX design.

When weak coupling and $\lambda \gg FWHM$ are assumed (which is true as $FWHM < 0.1nm$ and $\lambda \approx 1550nm$),

$$FWHM = \frac{\kappa^2\lambda^2}{2\pi^2 R n_{eff}} \qquad \textbf{Equation S3}$$

where κ is another coupling constant satisfying $|t|^2 + |\kappa|^2 = 1$.

With V_{switch} = 30 V, $FWHM = 2 \times 30 \times 0.4 = 24$ pm according to the sensitivity of 0.4 pm/V obtained from our previous publication*(51)*. With the simulated $n_{eff} = 1.662$ using the commercial software Lumerical (waveguide thickness = 0.4 μm, width = 1 μm), $|\kappa|^2$ can be calculated as 0.0131. Thus $|t| = 0.9934$.

On resonance where $\theta + \varphi_t = 2m\pi$ ($m = integer$), as required by Condition (A) in the main text, P_t must satisfy ER_{max} = 6 dB:

$$P_t = \frac{(\alpha-|t|)^2}{(1-\alpha|t|)^2} = 0.25 \qquad \textbf{Equation S4}$$

Solving Equation S4 with $|t| = 0.9934$, α =0.9803.

Since $|t|, \alpha, n_{eff}, and\ R$ are known, P_t can be plotted as a function of λ according to Equation S1, the result is shown in fig. S7.

When Condition (A) and Condition (B) are both satisfied, the sensitive range which is defined for the rate of change > 0.25, only covers a wavelength span of 48 pm. The 48 pm wavelength span corresponds to a voltage range of 48 / 0.4 = 120 V. Condition (C) is not satisfied.

Supplementary Text S2

Wavelet transform-enhanced DL

Wavelet transform can be used to detect events like anomalies, change points, and transients, and denoise and compress data, because it can analyse data at different time and frequency resolutions and decompose signals and images into their various components. Wavelet transform can obtain the characteristics of the frequency components and usually be used to analyse the signal with time-varying amplitude and frequency, which is widely applied in voice recognition, fault detection and diagnosis, *etc*.

Since the biometric information and control information presented in this work are instantaneous time-varying finite signals, wavelet transform might potentially bring some benefits in the data analysis. We apply the wavelet transform method to process our data for recognition. Specifically, we use the generalized Morse wavelet from Wavelet Toolbox (Matlab) to transform our original biometric signals. A typical original instantaneous time-varying finite biometric signal, the applied Morse Wavelet, and the resultant wavelet transform signal are shown in fig. S30. The biometric information (fig. S30A) is converted into a 2D map (fig. S30C) whose x-axis represents time and y-axis represents frequency. The 2D maps are then deemed as image data. We process all the 2208 data samples in the same way and turn them into image data. The representative 2D maps obtained from the instantaneous time-varying biometric signal from each of the 23 users are shown in fig. S31, illustrating their distinct features.

We then use these maps as the input for a CNN with 4 convolution layers and 4 max-pooling layers as shown in fig. S32, which has the same structure as the 1D-CNN we used for original biometric signals. The accuracy of recognition using signals after wavelet transformed ($O^{WT}{}_{bio}$) is 88.41%, which is comparable to the 89.49% accuracy of recognition using instantaneous time-varying signals ($O^{LP}{}_{bio}$). The similar accuracy between using wavelet transform and using the instantaneous time-varying signals could be possibly attributed to the lack of frequency information in the biometric signal. The frequencies of all the biometric signals are very low, and thus do not provide significant features to classify different users.

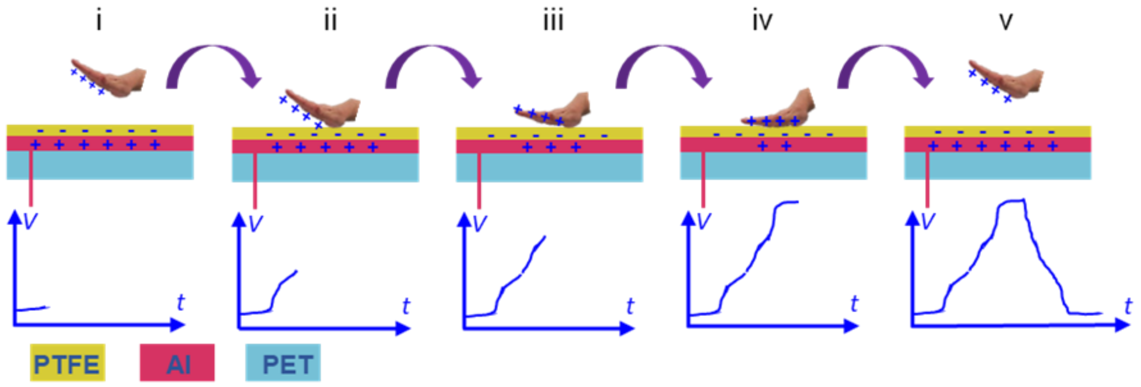

Fig. S1. Triboelectric contact-separation-based single-electrode mode working mechanism. The central ellipse of TEB scanner works in the triboelectric contact-separation-based single-electrode mode. Electric charges are generated upon human interaction. The finger and the PTFE surface have positive and negative charges respectively. When the palm is far away from the PTFE surface (**State (i)**), the voltmeter detects a low voltage. From **State (i)** to **State (iv)**, the palm gradually comes into full contact with the PTFE surface, leading to a voltage increase, because the positive charges on the palm will neutralize the negative charges on the PTFE. At **State (v)**, the palm leaves the TEB scanner. Consequently, the voltage drops to the lowest value.

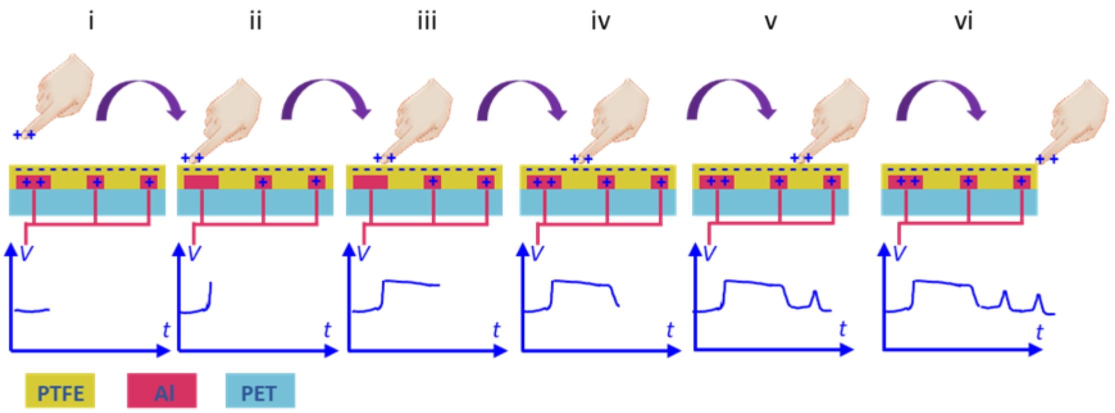

Fig. S2. Triboelectric sliding-based single-electrode mode working mechanism.
The eight digitated electrodes protruding from the central ellipse work in the triboelectric sliding-based single-electrode mode. The electrode encoded by **'010'** is taken as an example without loss of generality. Initially, the finger and the PTFE surface have positive and negative charges respectively. When the finger is far away from the PTFE surface (**State (i)**), the voltmeter detects a low voltage. When the finger touches the central ellipse (**State (ii)**), some negative charges on the ellipse will be neutralized, causing the voltage to rise. Before the finger leaves the central ellipse (from **State (ii)** to **State (iii)**), the amount of neutralized charge maintains unchanged, resulting in a stable high voltage. This steady high voltage represents **'Start'**. At **State (iv)**, the finger is in the middle of two electrodes and has a small electrostatic induction. Therefore, the voltage falls back to the low level, leading to a 'ZERO'. From **State (iv)** to **State (v)**, the finger sweeps the middle electrode bar and arrives amid the middle electrode bar and the last electrode bar. As explained above, high voltage levels will only be caused by fingers right on top of the electrode. Thus, a 'ONE' is obtained followed by another 'ZERO' between **State (iv)** to **State (v)**. Finally, as the finger sweeps the last electrode bar (**State (vi)**), the last 'ONE' representing the 'End' will be generated. The final encoded information is then '010'. The valid count of 'ONE' is determined by a voltage threshold, while that of 'ZERO' is determined by the duration of the low voltage level. The effectiveness of this method has been proven in ref*(38)*.

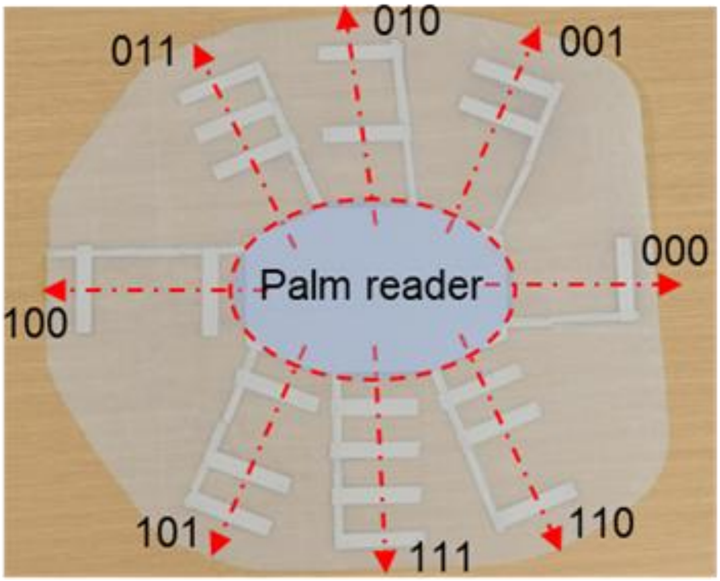

Fig. S3. Optical image of the manufactured TEB scanner.

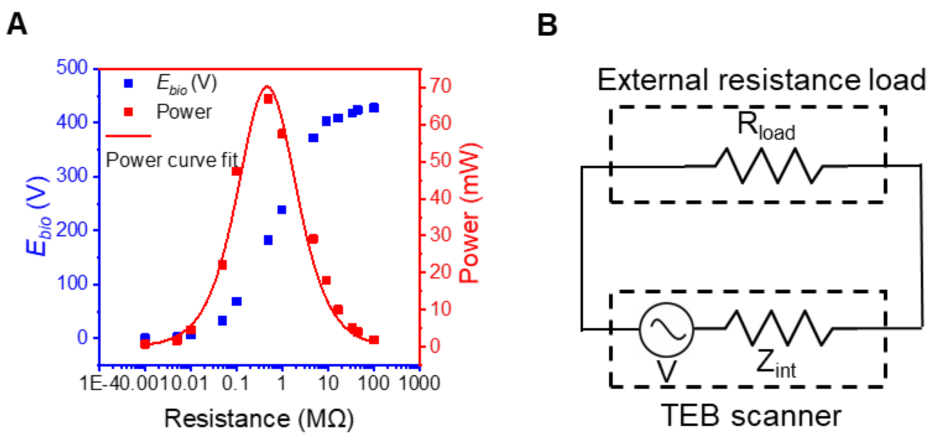

Fig. S4. Calculate the internal impedance of TEB scanner.
(A) Voltage and power output of TEB scanner under different external resistances. **(B)** Equivalent circuit model of TEB scanner with external resistance loads.

The output voltage and power are measured with different external resistance loads in the case of human palm interaction (fig. S4A). Using a simple electrical model where TEB scanner is a voltage source with internal impedance Z_{int} and the load resistance is R_{load} (fig. S4B), the output power can be expressed as:

$$\boldsymbol{P} = \left(\frac{\boldsymbol{V}}{\boldsymbol{Z_{int}} + \boldsymbol{R_{load}}}\right)^2 \times \boldsymbol{R_{load}}, \qquad \textbf{Equation S5}$$

Fitting the power curve in fig. S4A using Equation S5, Z_{int} and V can be extracted as coefficients. The adj. R-square of the fitting is 0.974. Z_{int} and V are 0.467 MΩ and 11.44 (corresponding to 361.76 V) respectively. Thus, the maximum instantaneous power of $V^2/4Z_{int}$ = 70.1 mW is obtained.

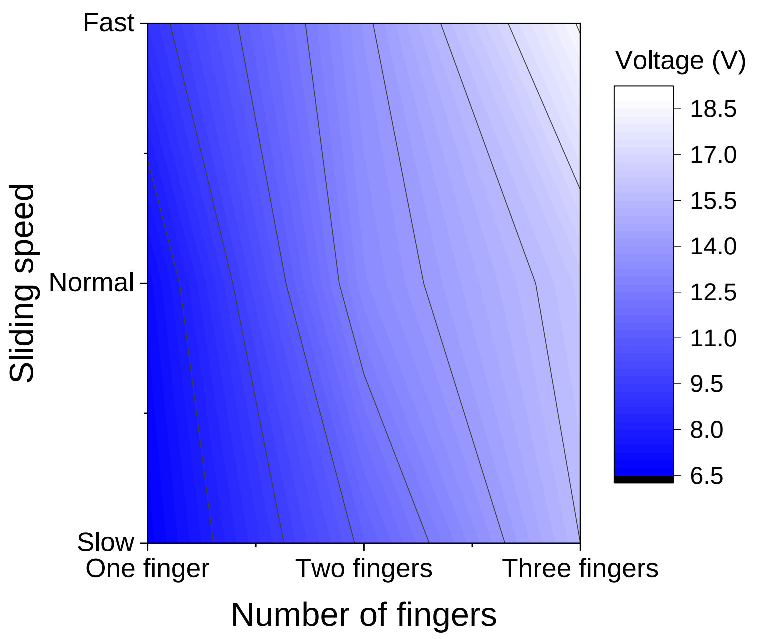

Fig. S5. Dependence of the voltage of self-generated $E_{control}$ on finger sweep speed and the number of fingers.

A

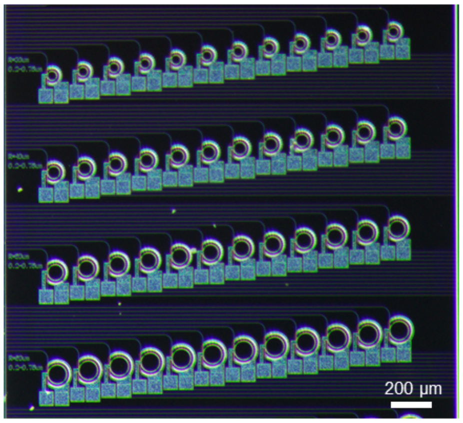

B

Radius (µm)	Min. gap (µm)	Max. gap (µm)	Gap step (µm)	FSR (nm)
30	0.2	0.75	0.05	6.01
40	0.2	0.75	0.05	4.56
50	0.2	0.75	0.05	3.62
60	0.2	0.75	0.05	3.08

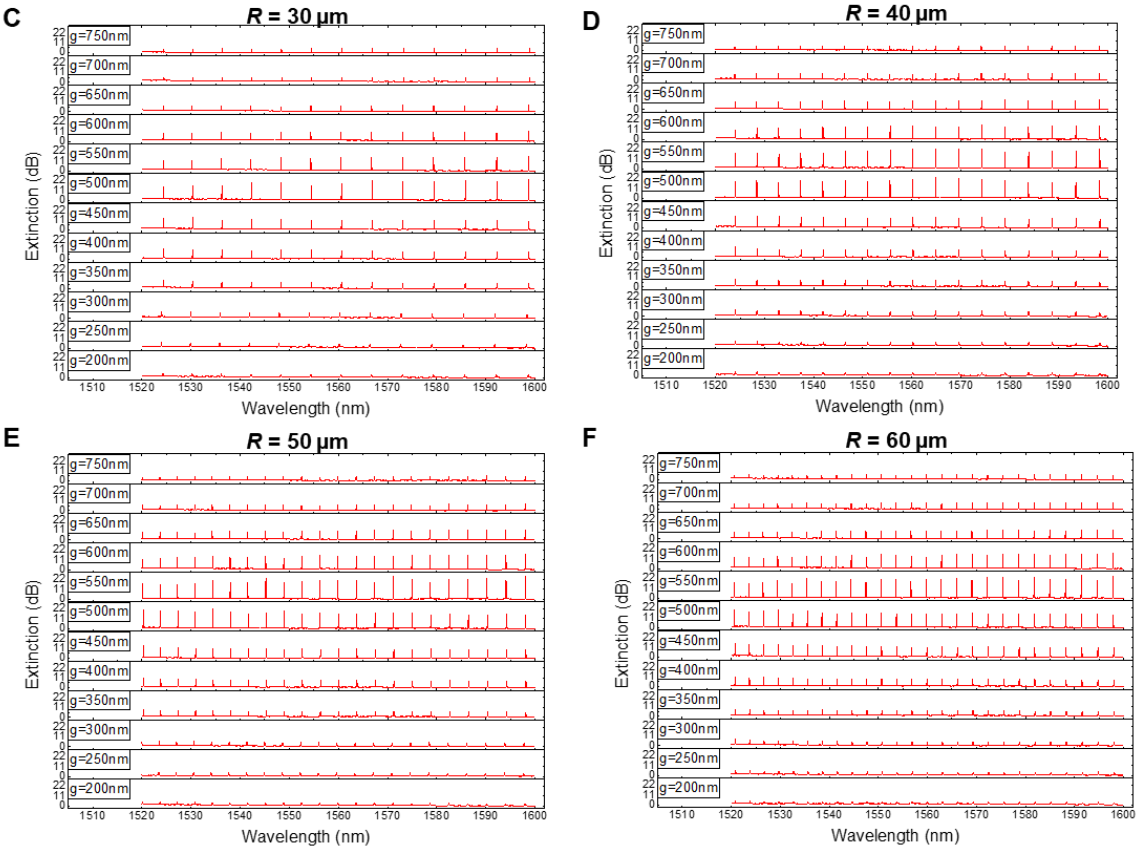

Fig. S6. Spectra of 48 MRRs which are formed by the combination of 4 different Rs and 12 different gs.

(A) Optical image. **(B)** Design parameters of *R* and *g*, and measured FSR. **(C to F)** , Measured spectrum of MRRs with **(C)** R = 30 µm, **(D)** R = 40 µm, **(E)** R = 50 µm, **(F)** R = 60 µm. FSR, free spectral range.

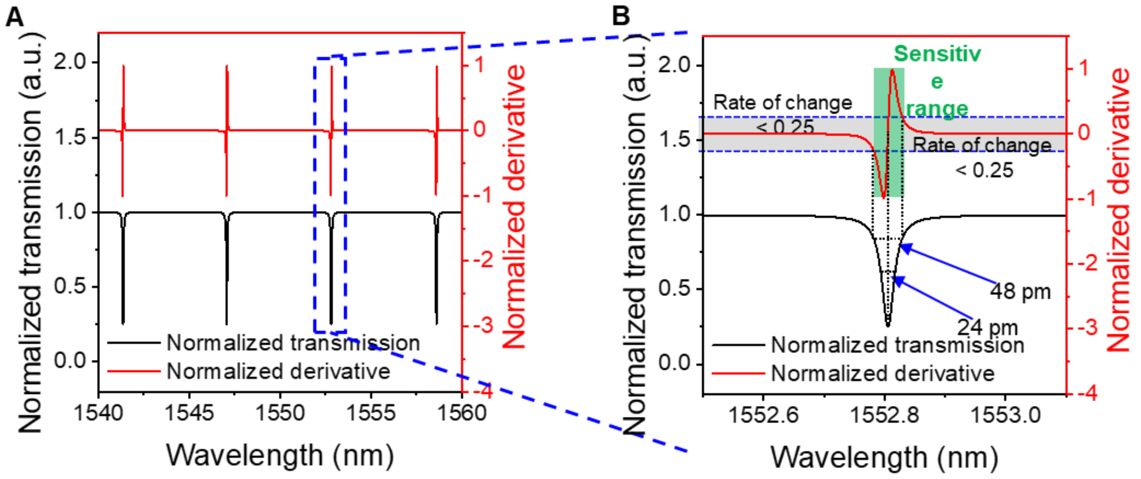

Fig. S7. Calculated transmission of a single MRR with satisfying ER_{max} = 6 dB and V_{switch} = 30 V. The requirements are equivalent as $P_{t\_minimum}$ = 0.25 and FWHM = 24 pm.

(A) The calculated spectrum showing $P_{t\_minimum}$ = 0.25. **(B)** The zoom-in trough showing FWHM = 24 pm.

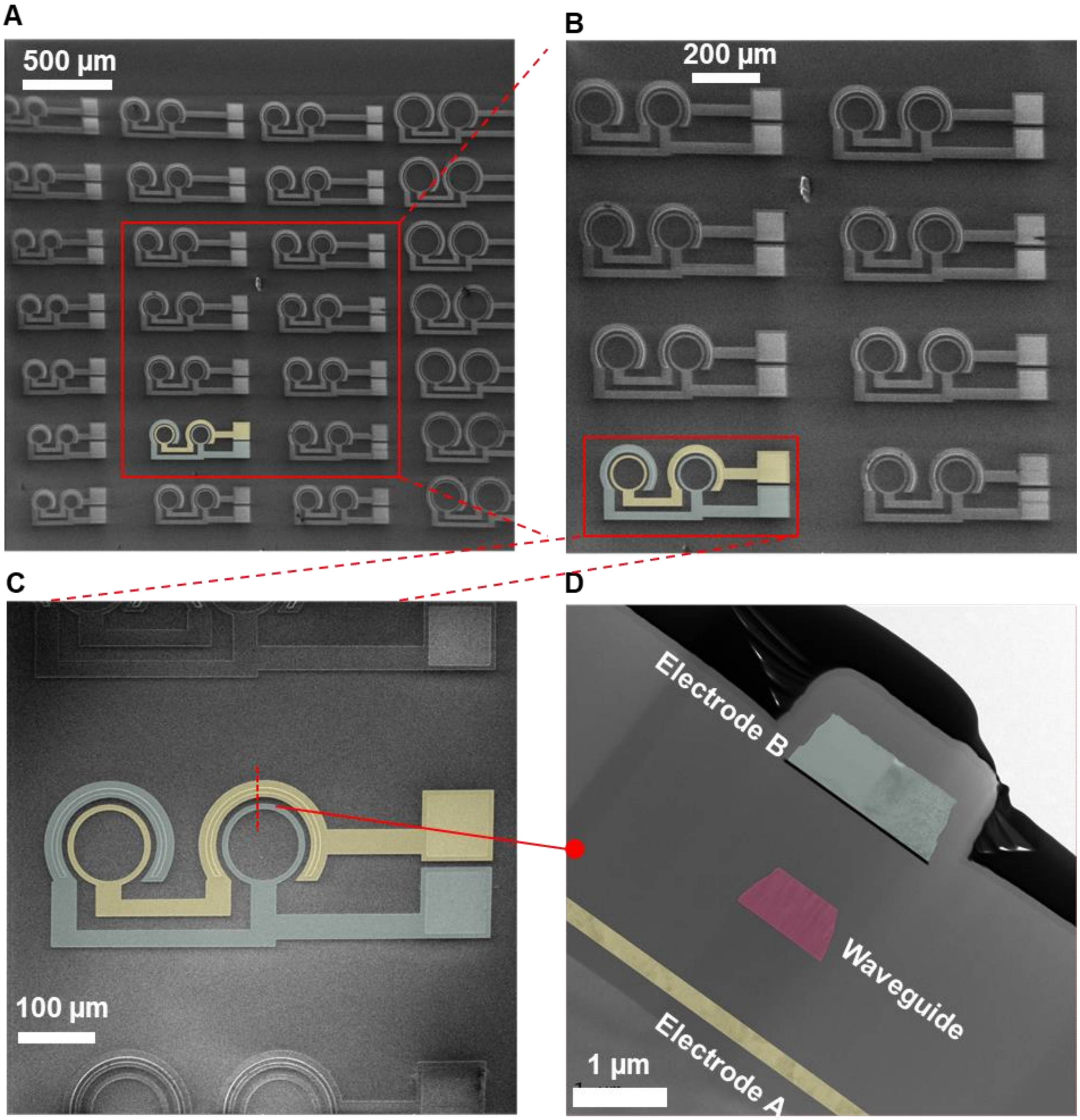

Fig. S8. False colored SEM and TEM images of manufactured CMRRs.
(A to C) SEM images under different magnifications. **(D)** TEM image of the cross-section labeled in **(C)**.

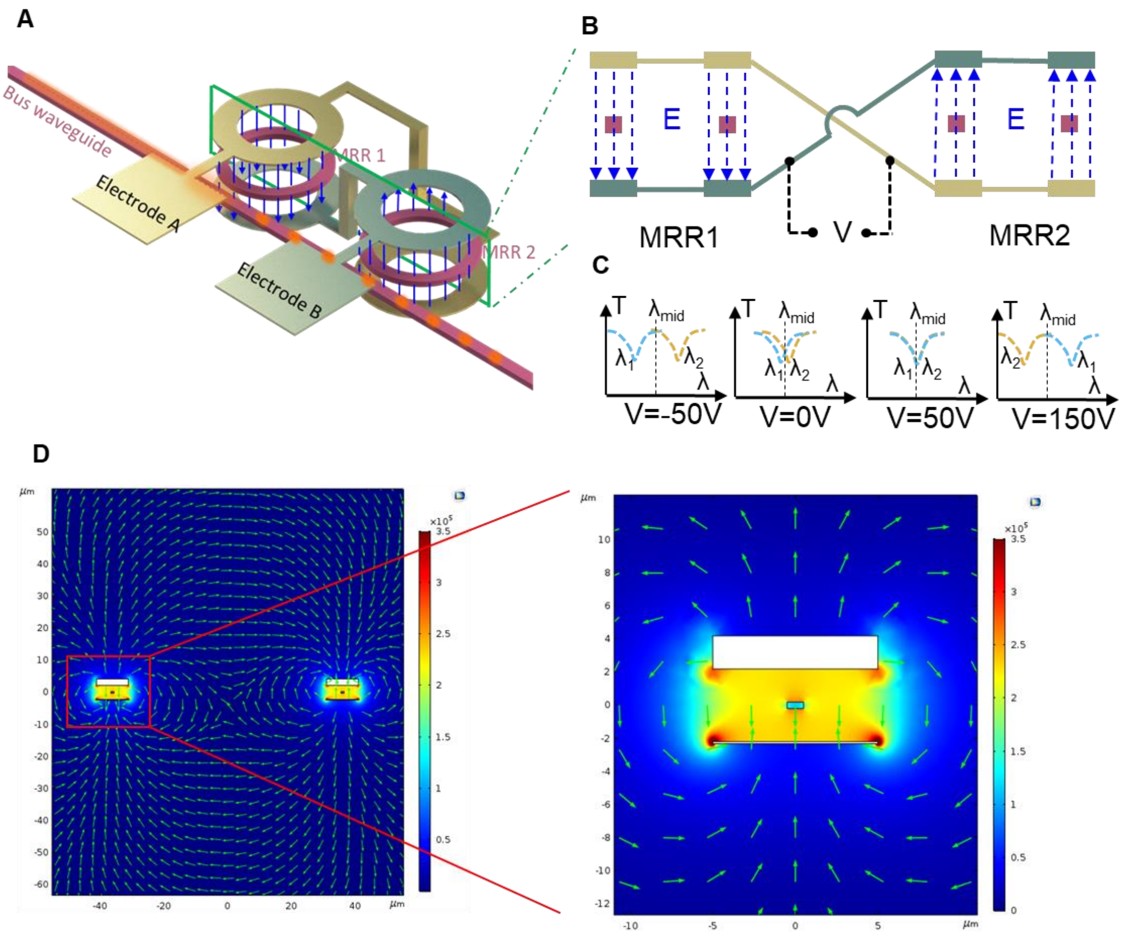

Fig. S9. CMRR push-pull mechanism.

(A) Device configuration. **(B)** Cross-section of the CMRR. **(C)** Symmetric tuning with respect to λ_{mid}. **(D)** Electric filed distribution simulated by COMSOL.

Fig. S9B shows the CMRR cross-section highlighted in the green box in fig. S9A. The bottom electrode of the left MRR is connected to the top electrode of the right MRR and vice versa. Thus, when a voltage *V* is applied, opposite electric fields *E* with the same magnitude will penetrate through each MRR, leading to opposite resonant wavelength shift ($\pm \Delta\lambda$). As shown in fig. S9C, resonant wavelength λ_1 = 1551.4596 nm of MRR1 and λ_2 = 1551.4916 nm of MRR2 are separated by a small value of 32 pm as fabricated. It is worth noting that the 32 pm difference is designed such that it can be compensated by around 50 V, *i.e.* $(\lambda_1+\Delta\lambda) = (\lambda_2 - \Delta\lambda) = \lambda_{mid} = (\lambda_1+\lambda_2)/2$ = 1551.4756 nm at 50 V. As a result, the optical transmission O_{bio} is symmetric *w.r.t.* the voltage span from -50 V to 150 V. The above discussion is further verified by COMSOL simulation results shown in fig. S9D**.** And it confirms that the electric fields in individual MRRs are vertical and not affected by adjacent MRR.

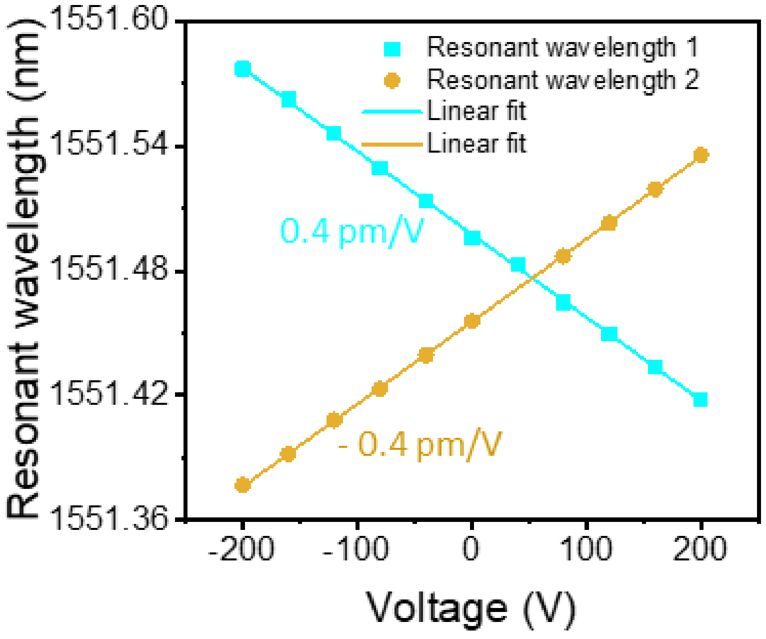

Fig. S10. Opposite linear resonant wavelength tuning with sensitivity 0.4 pm/V in BOIMUX.

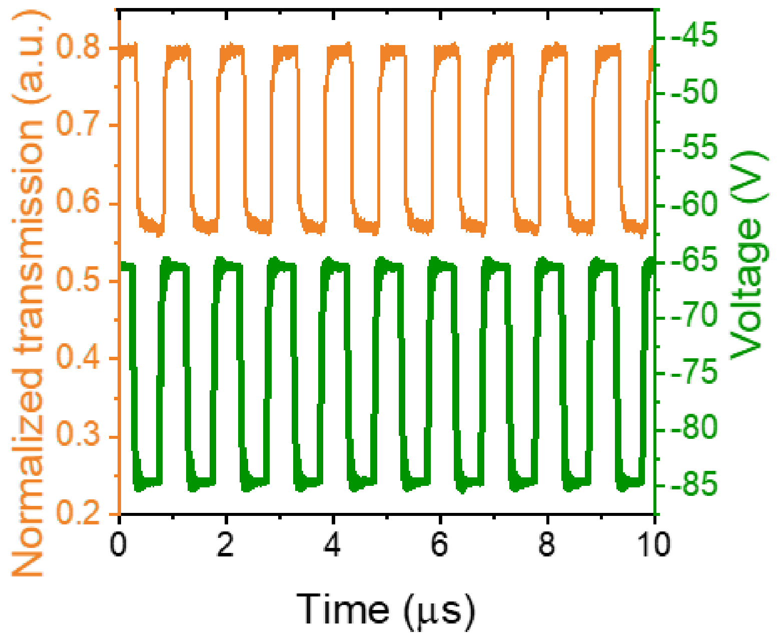

Fig. S11. AC tuning of BOIMUX using a 1 MHz square wave with 20 V V_{pp} and -75 V V_{offset}.

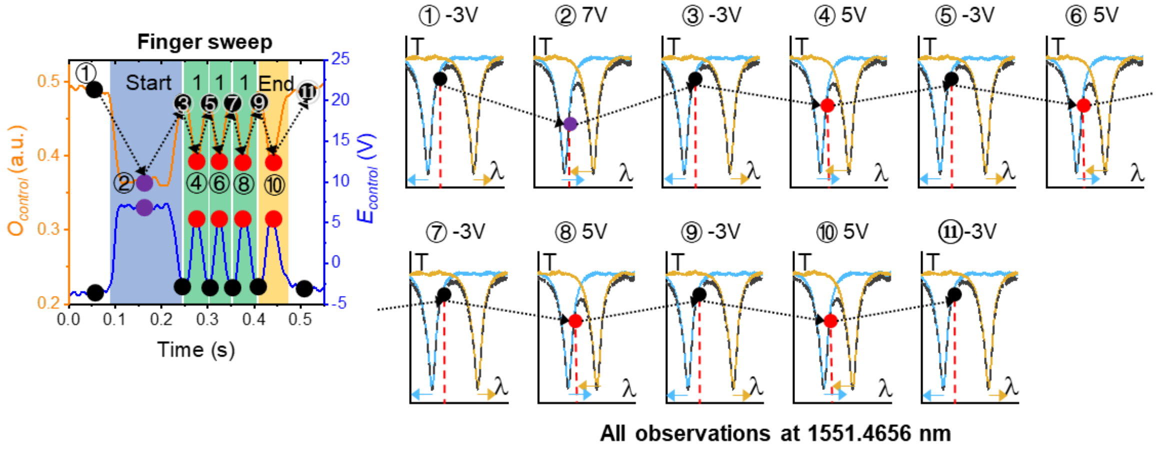

Fig. S12. Explanation of the observed $E_{control}$ to $O_{control}$ conversion using schematic spectra of $O_{control}$ under different $E_{control}$s at different states.

The control signal '111' is taken as the example without loss of generality. All observations are at 1551.4656 nm. State ① is before the finger touches the TEB scanner. $E_{control}$ is at -3 V. The blue resonance λ_1 (blue spectrum) is at 1551.4556 nm. Thus, a high $O_{control}$ around 0.5 is observed. When the finger touches the middle ellipse of the TEB scanner (State ②), $E_{control}$ rises from -3 V to 7 V (the rise is explained fig. S2) leading to the redshift of λ_1 to 1551.4604 nm. A low $O_{control}$ of around 0.36 is observed. Next, from State ② to State ③, the finger leaves the central ellipse. $E_{control}$ falls back to -3 V, and $O_{control}$ rises back to around 0.45. From State ③ to State ⑤, the finger sweeps across the first bar of '111'. $E_{control}$ rises from State ③ to State ④, and drops from State ④ to State ⑤. Accordingly, $O_{control}$ drops from State ③ to State ④, and rises from State ④ to State ⑤. The finger then sweeps across the second bar, the third bar, and the last bar, corresponding to State ⑤ to ⑦, ⑦ to ⑨, and ⑨ to ⑪, respectively. They can be explained similarly as the process from State ③ to State ⑤. The other seven three-digit binary codes can be understood in a similar way.

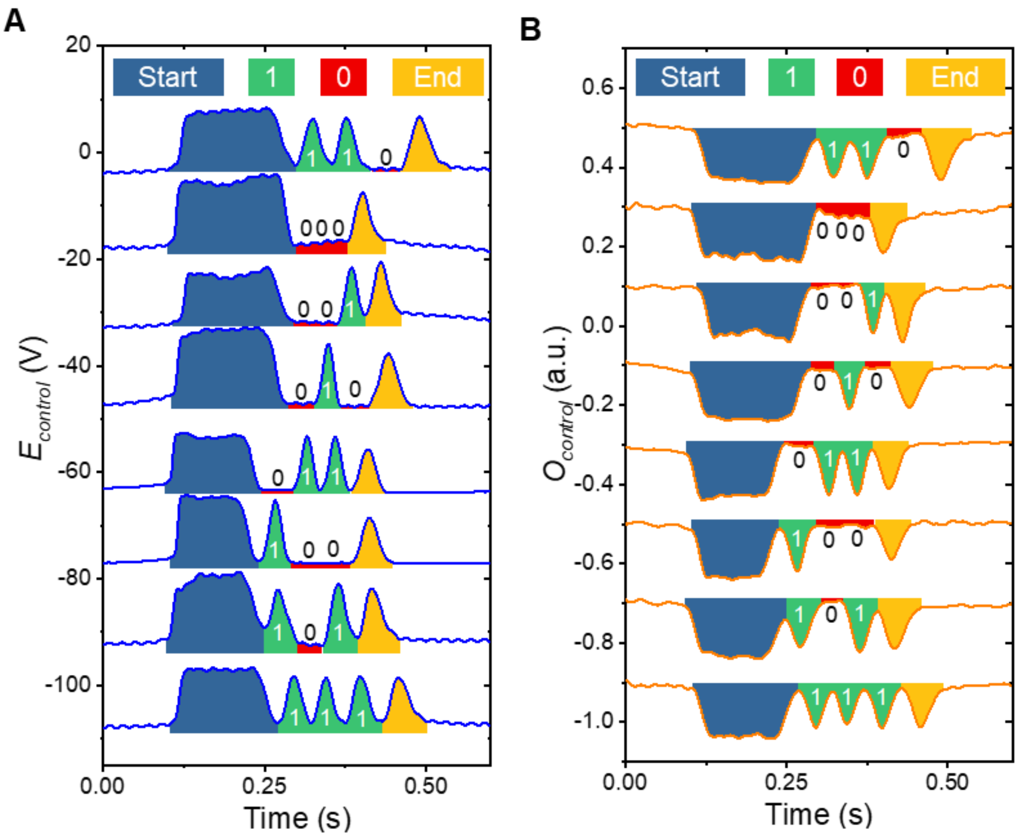

Fig. S13. Conversion from $E_{control}$ to $O_{control}$ when a finger sweeps across eight different digitized electrodes.

(A) $E_{control}$, **(B)** $O_{control}$.

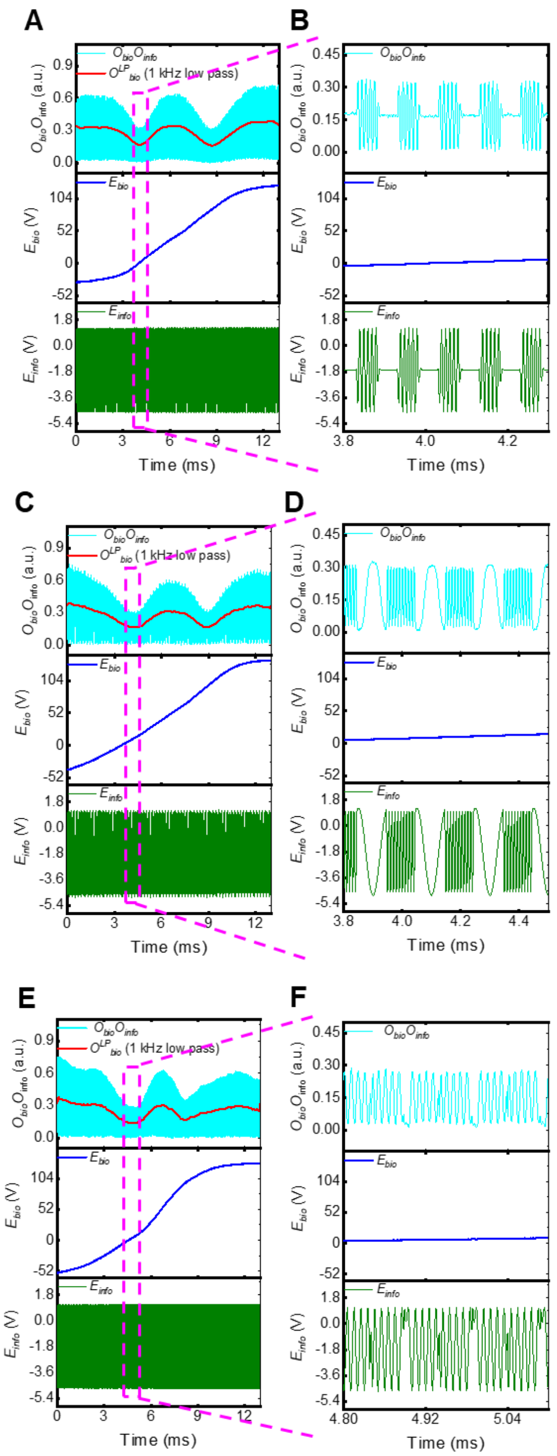

Fig. S14. Multiplexing biometric information and modulated digital information.

Multiplexed $O_{bio}O_{info}$ (cyan) generated by using E_{bio} (blue) to modulate O_{info} (green) and the corresponding extracted biometric information O^{LP}_{bio} (red) using a 1 kHz FFT low-pass filter. The digital information is 100 kHz and the modulation frequency is 10 kHz. **(A** and **B),** AM. **(C and D),** FM. **(E and F),** PM. AM, amplitude-modulated; FM, frequency-modulated; PM, phase-modulated.

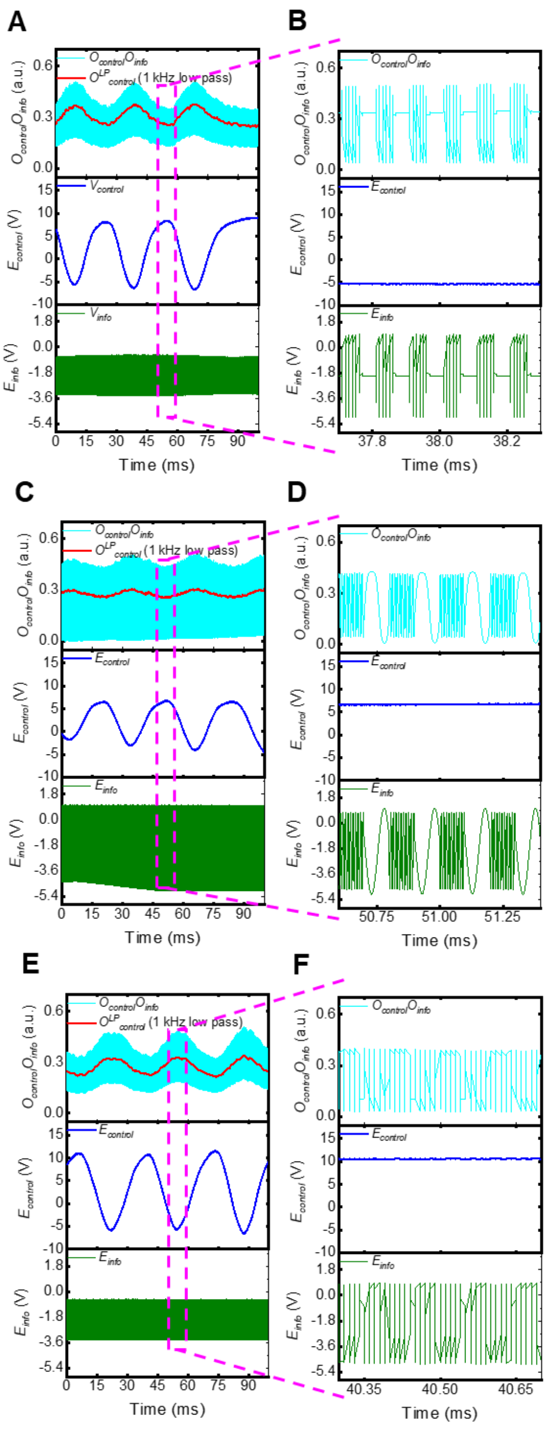

Fig. S15. Multiplexing finger sweep control information and modulated digital information. Multiplexed $O_{control}O_{info}$ (cyan) generated by using $E_{control}$ (blue) to modulate O_{info} (green) and the corresponding extracted biometric information $O^{LP}_{control}$ (red) using a 1 kHz FFT low-pass filter. The digital information is 100 kHz and the modulation frequency is 10 kHz. **(A and B),** AM. **(C and D),** FM. **(E and F),** PM. AM, amplitude-modulated; FM, frequency-modulated; PM, phase-modulated.

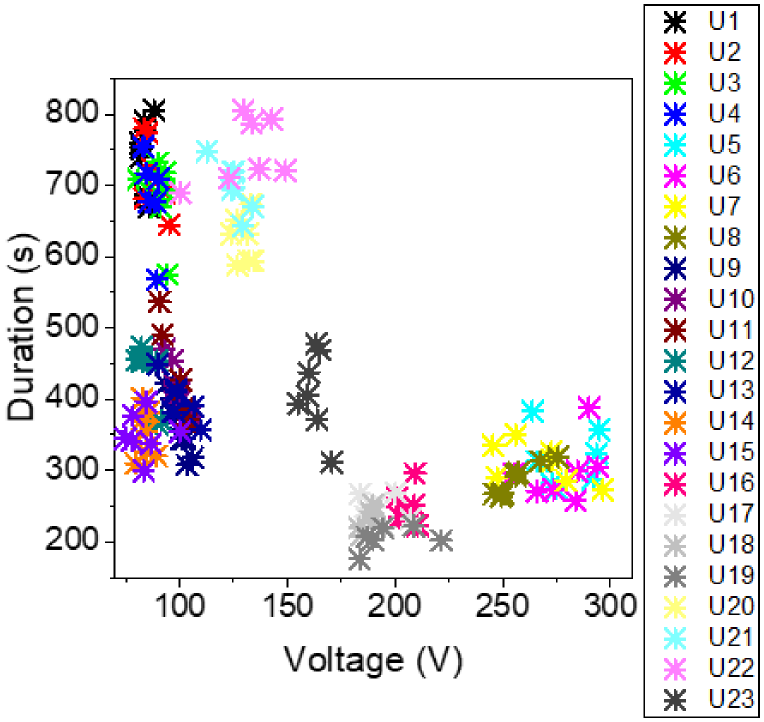

Fig. S16. Distinguish 23 users using the voltage span and duration of E_{bio}. Each user contains 7 samples.

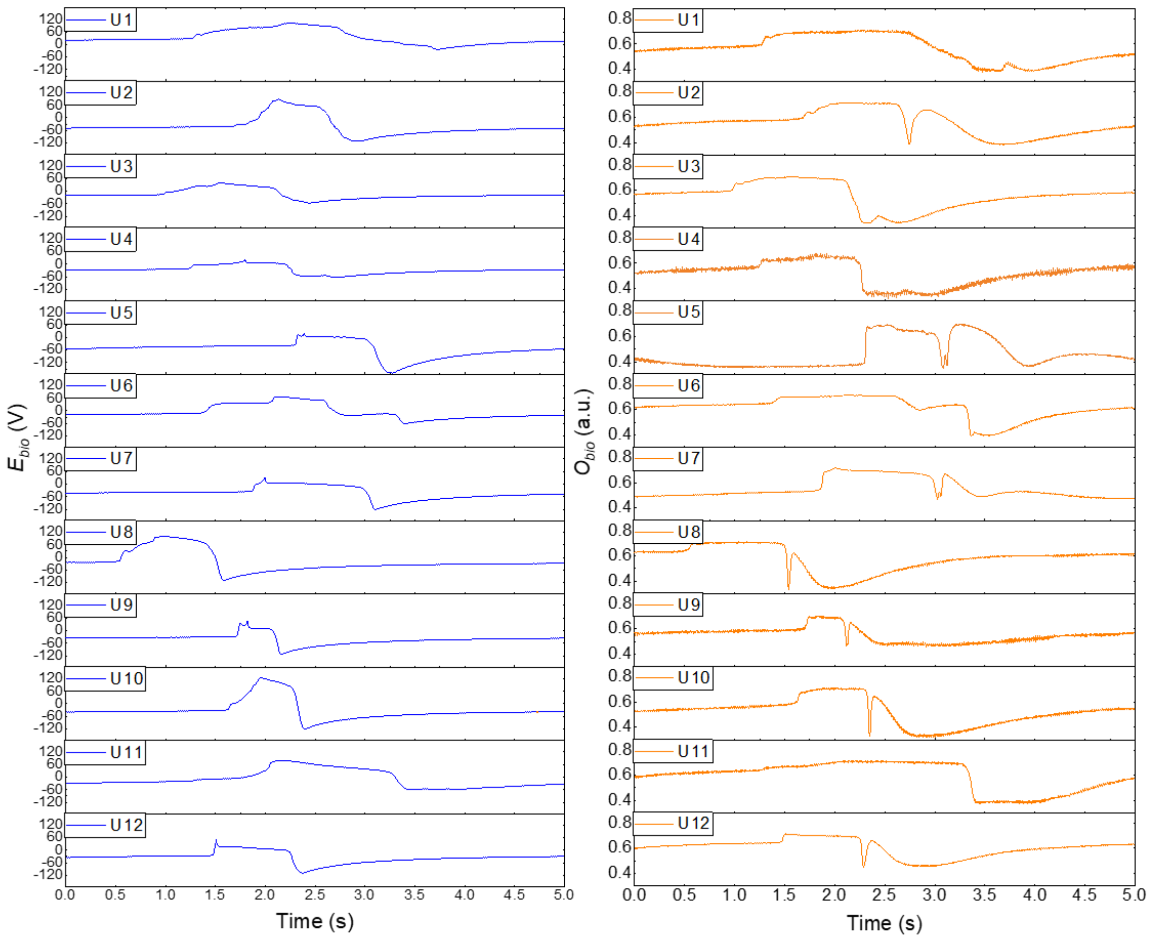

Fig. S17. Typical biometric information E_{bio} (blue) and corresponding O_{bio} (orange) of User 1 to User 12.

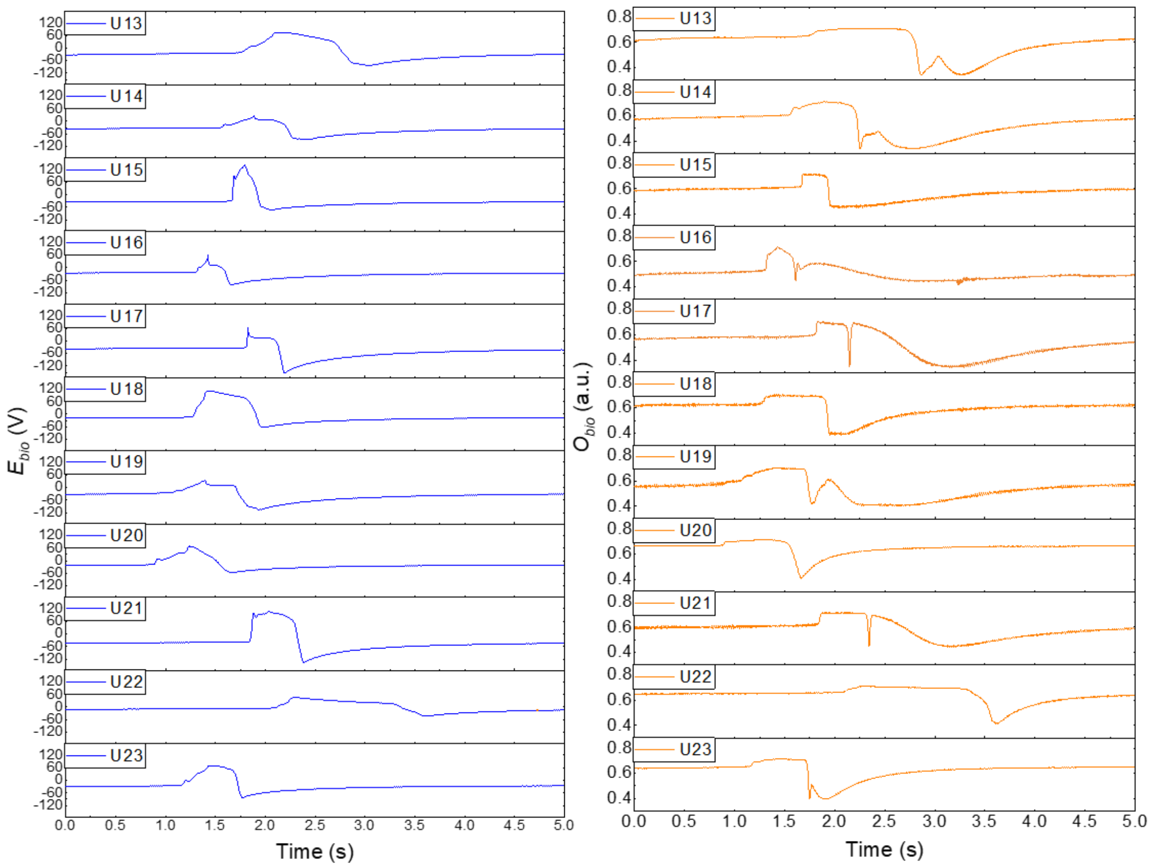

Fig. S18. Typical biometric information E_{bio} (blue) and corresponding O_{bio} (orange) of User 13 to User 23.

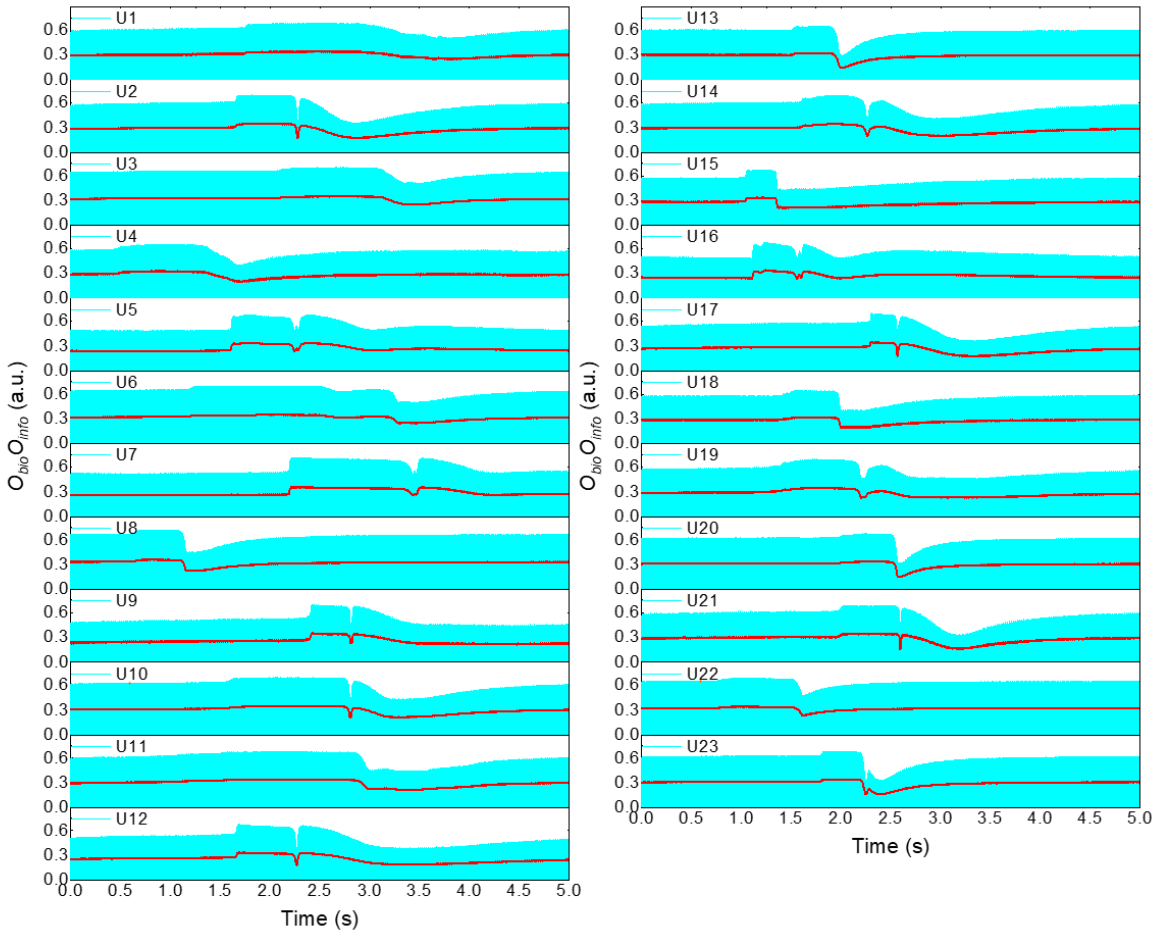

Fig. S19. Typical biometric information $O_{bio}O_{info}$ (cyan) and corresponding $O^{LP}{}_{bio}$ (red) of User 1 to User 23.

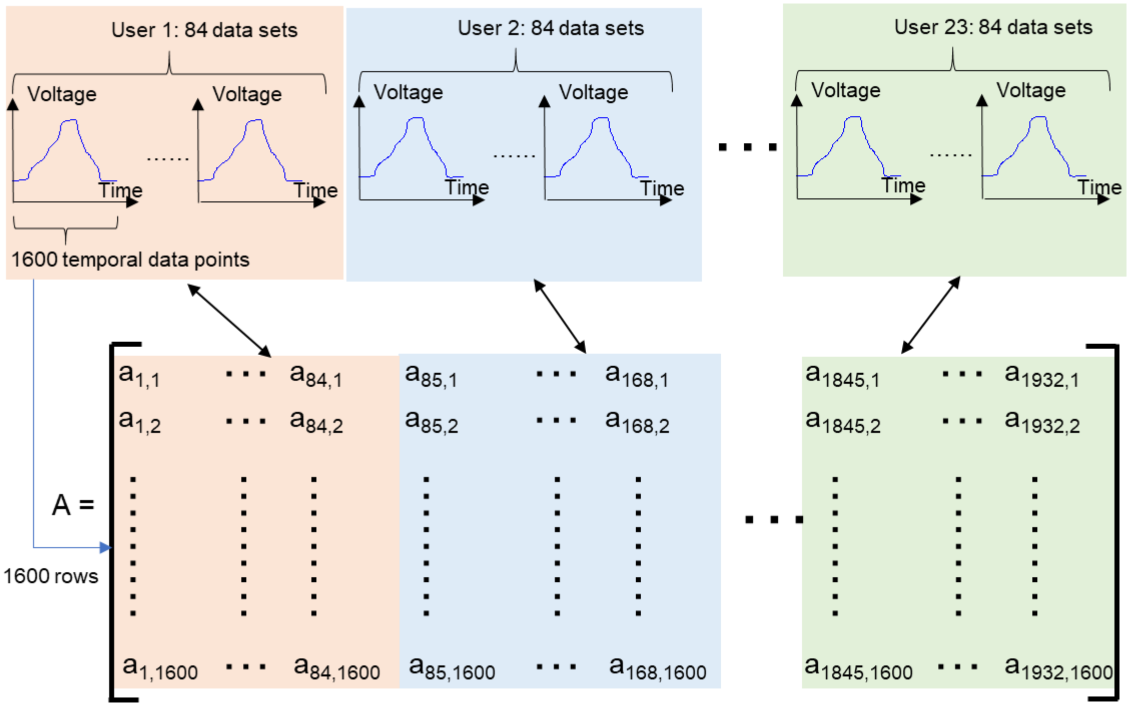

Fig. S20. Construction of 1600 by 1932 matrices for 2D-PCA using data collected from 23 users each with 84 data sets.

Every data set contains 1600 data points. E_{bio} is used graphically without loss of generality.

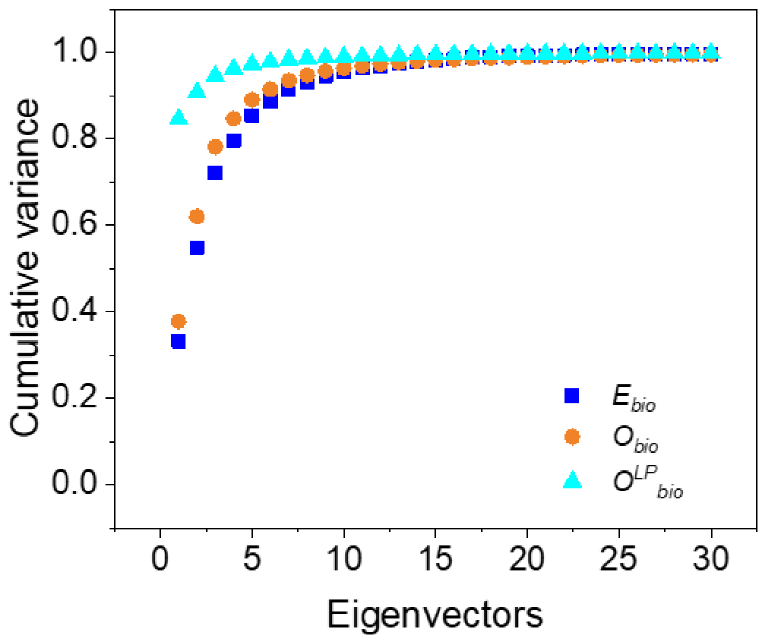

Fig. S21. Cumulative variance of 2D-PCA results.

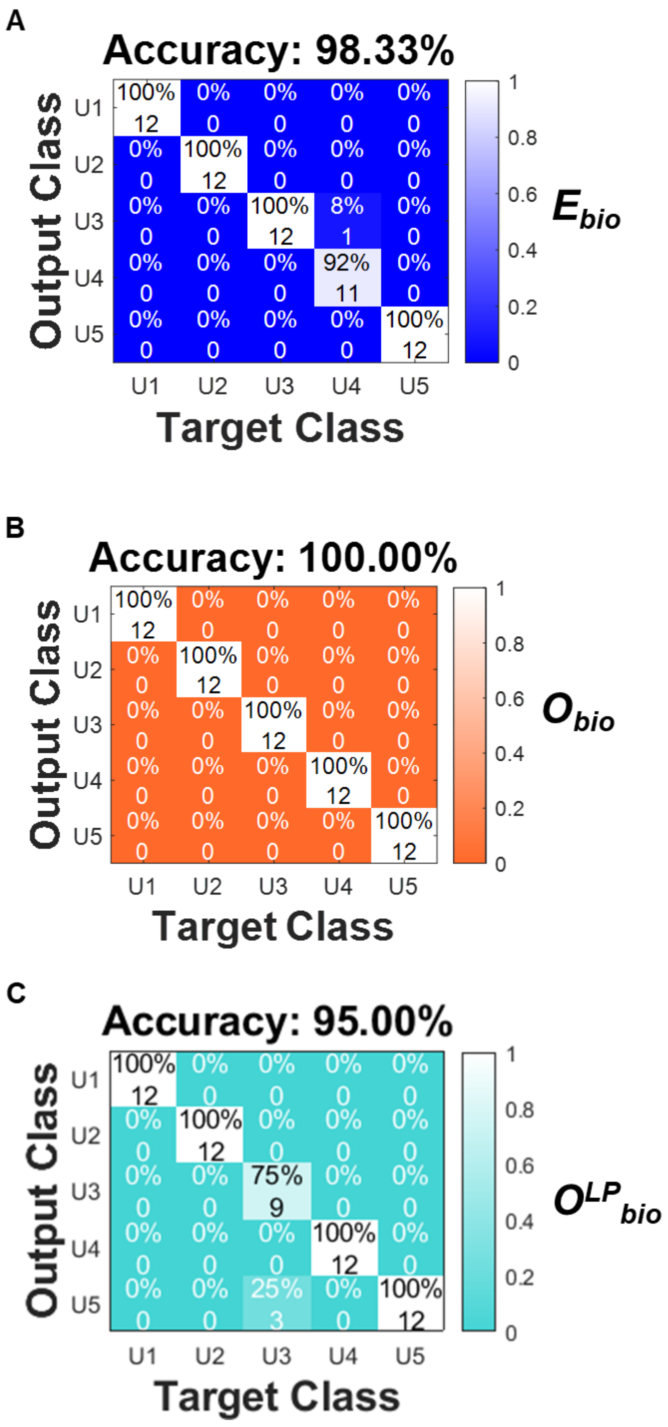

Fig. S22. Confusion map of the prediction with E_{bio} (blue), O_{bio} (orange), and O^{LP}_{bio} (cyan) generated by 5 different users.

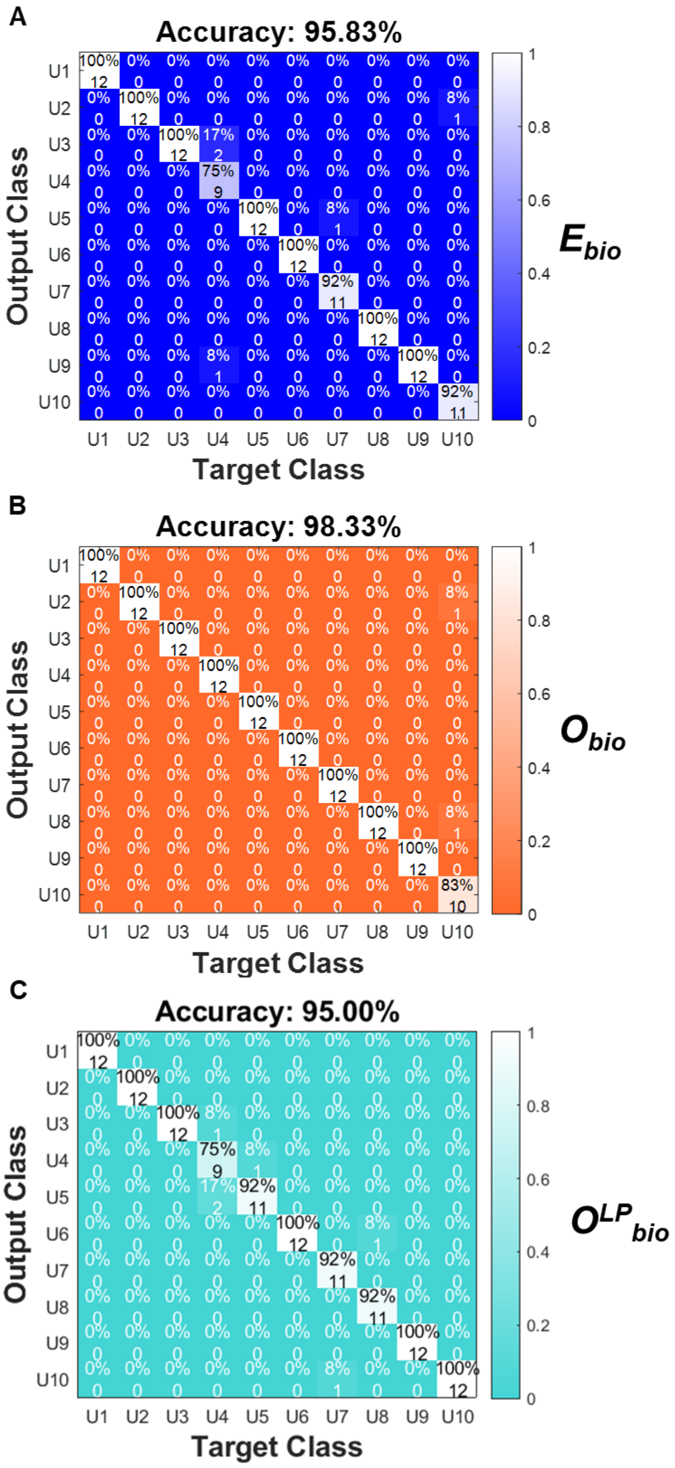

Fig. S23. Confusion map of the prediction with E_{bio} (blue), O_{bio} (orange), and O^{LP}_{bio} (cyan) generated by 10 different users.

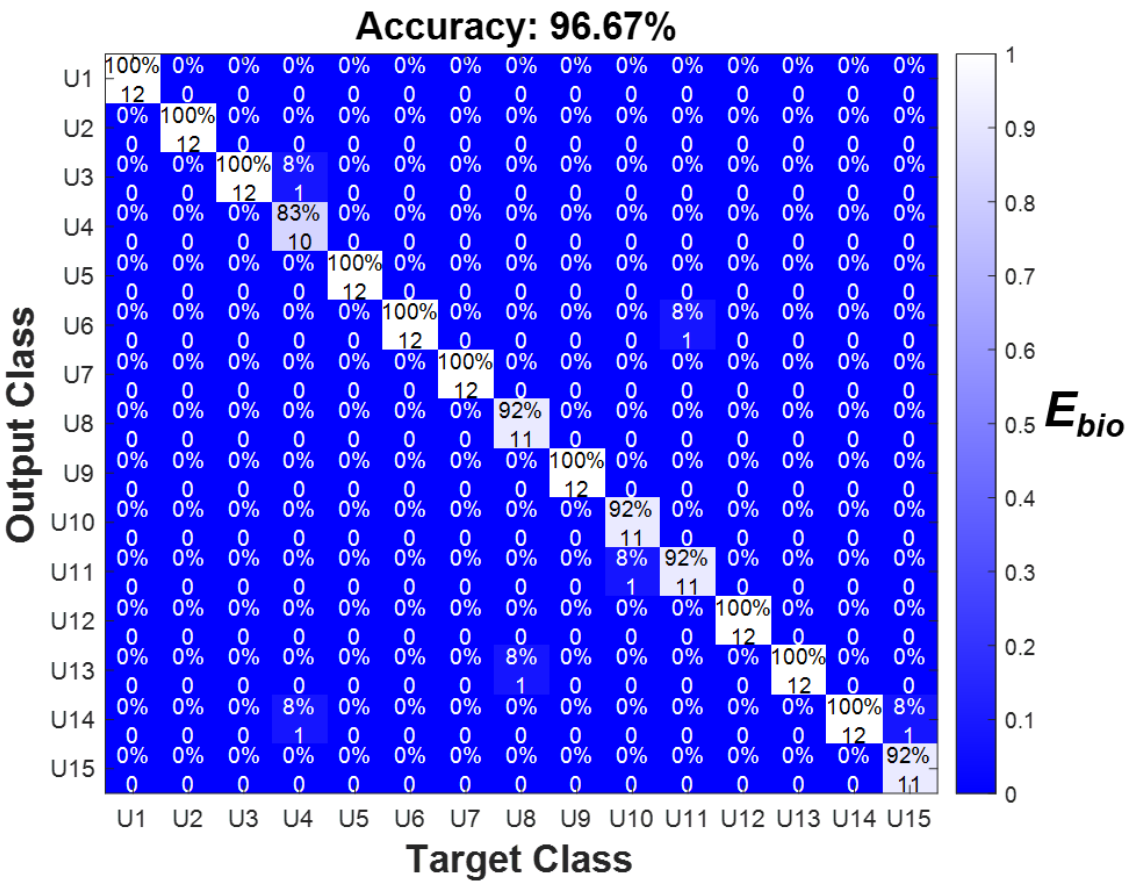

Fig. S24. Confusion map of the prediction with E_{bio} generated by 15 different users.

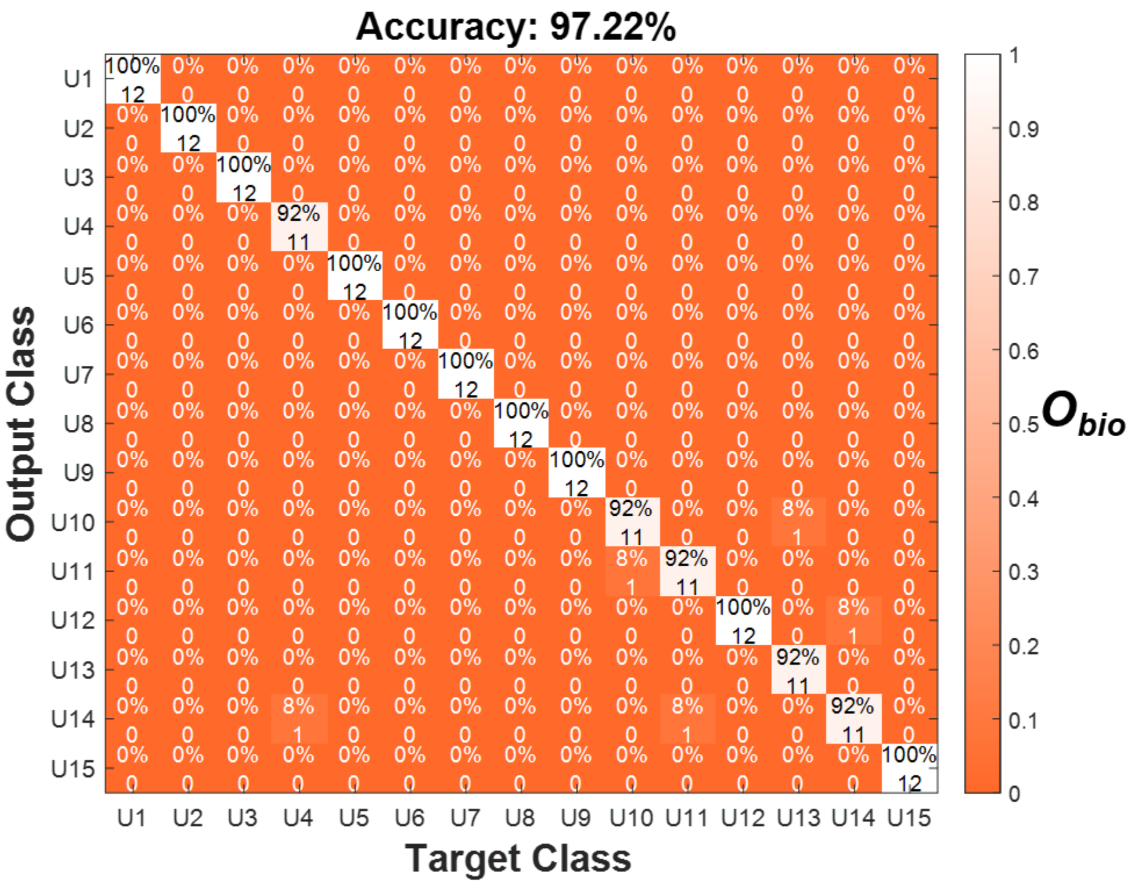

Fig. S25. Confusion map of the prediction with O_{bio} generated by 15 different users.

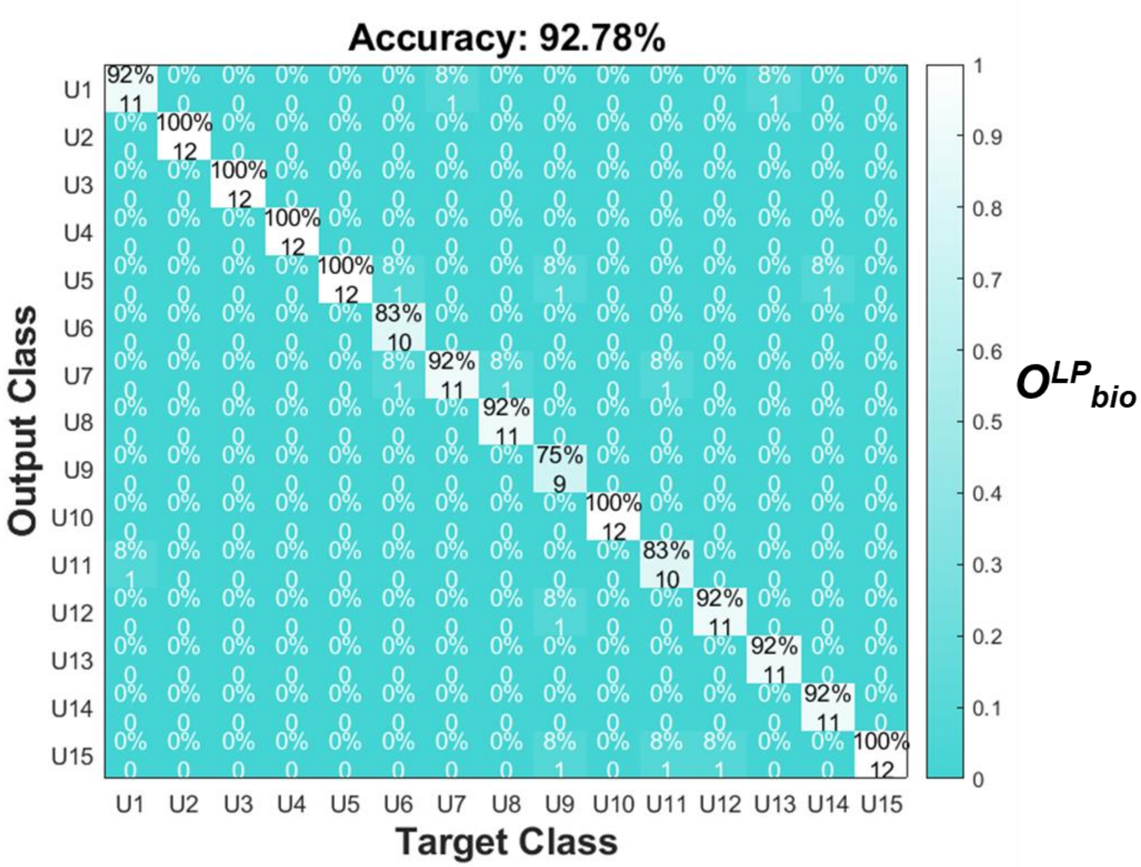

Fig. S26. Confusion map of the prediction with $O^{LP}{}_{bio}$ generated by 15 different users.

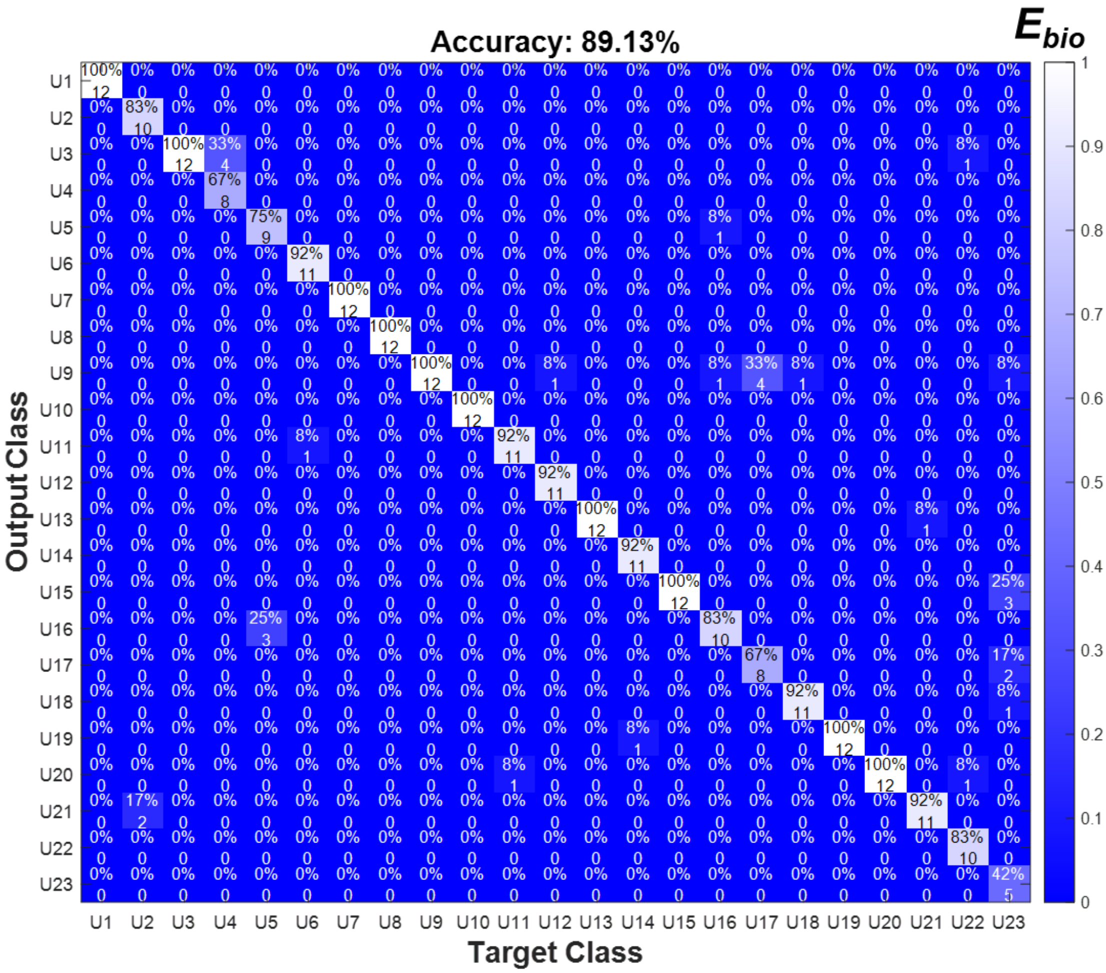

Fig. S27. Confusion map of the prediction with E_{bio} generated by 23 different users.

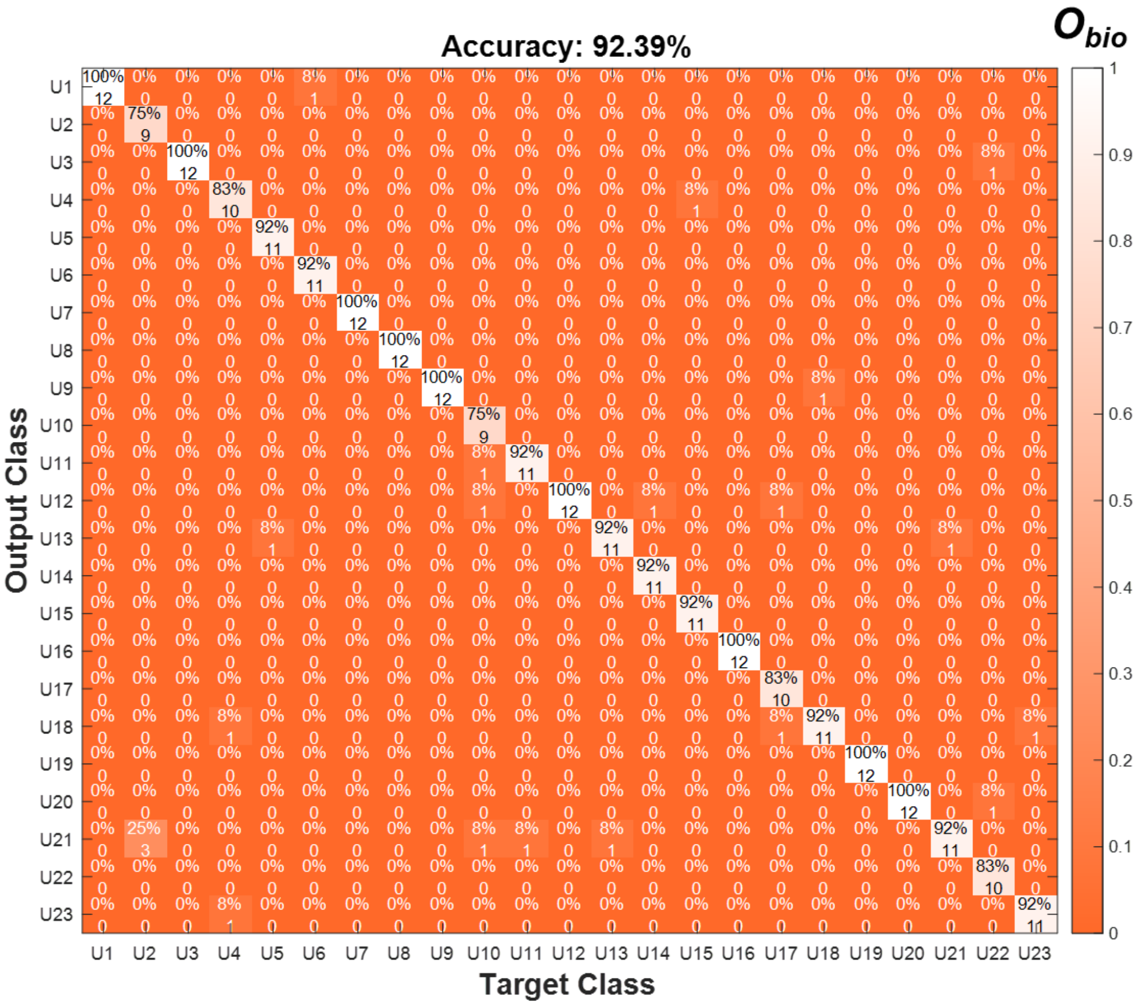

Fig. S28. Confusion map of the prediction with O_{bio} generated by 23 different users.

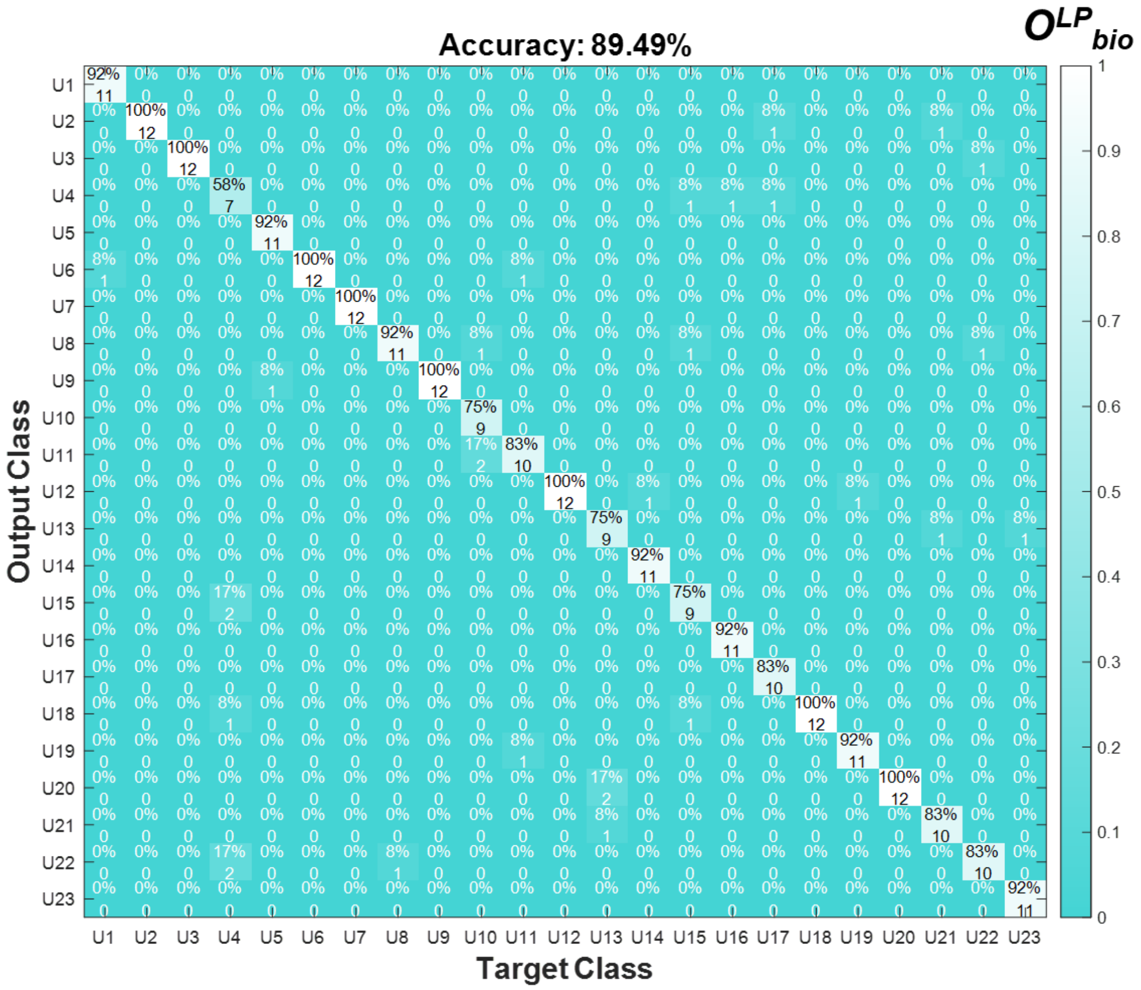

Fig. S29. Confusion map of the prediction with $O^{LP}{}_{bio}$ generated by 23 different users.

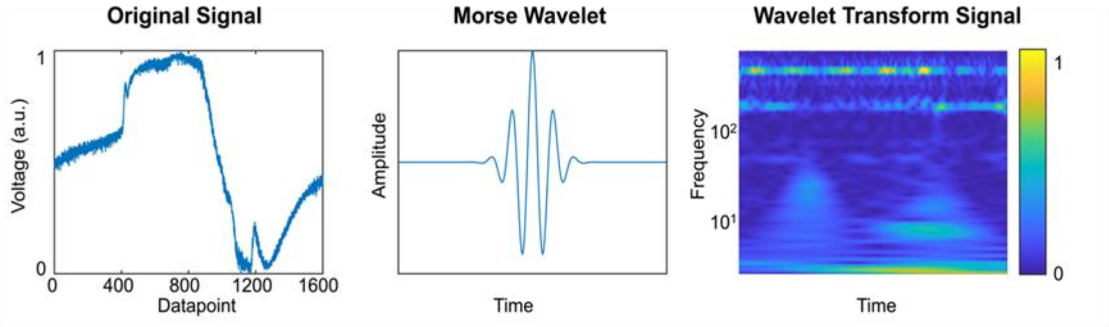

Fig. S30. Use a Morse wavelet to convert biometric information into a 2D map containing both time and frequency information.

A typical **(A)** original instantaneous time-varying finite biometric signal, **(B)** the applied Morse wavelet, **(C)** the obtained wavelet transform signal that contains both time and frequency information.

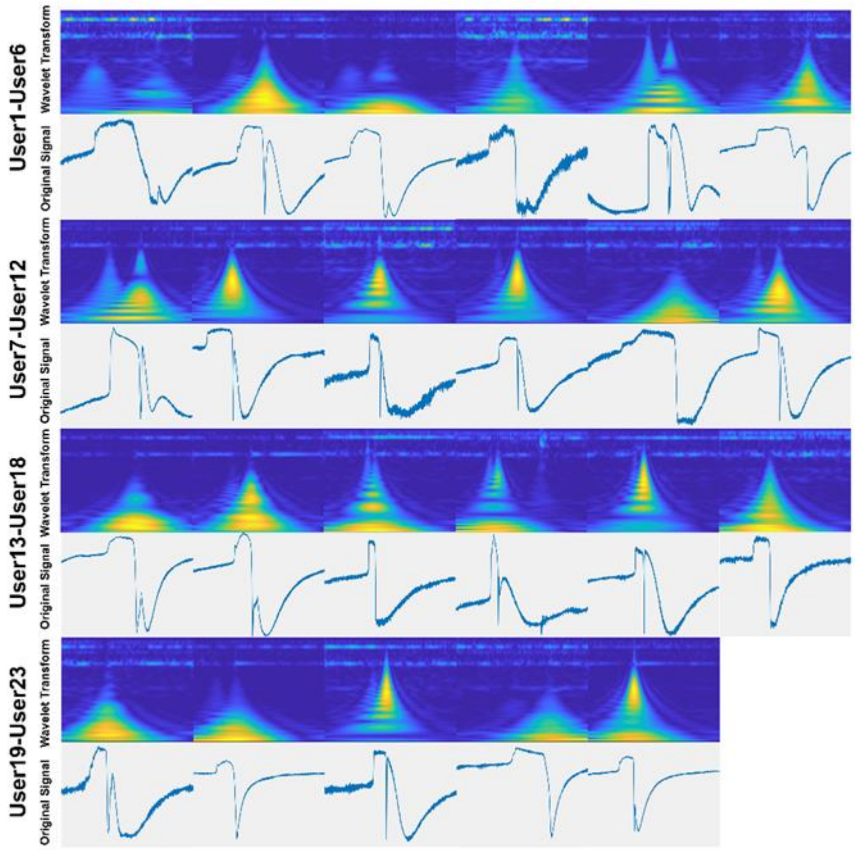

Fig. S31. Representative 2D maps obtained from the instantaneous time-varying biometric signal from each of the 23 users after wavelet transform.

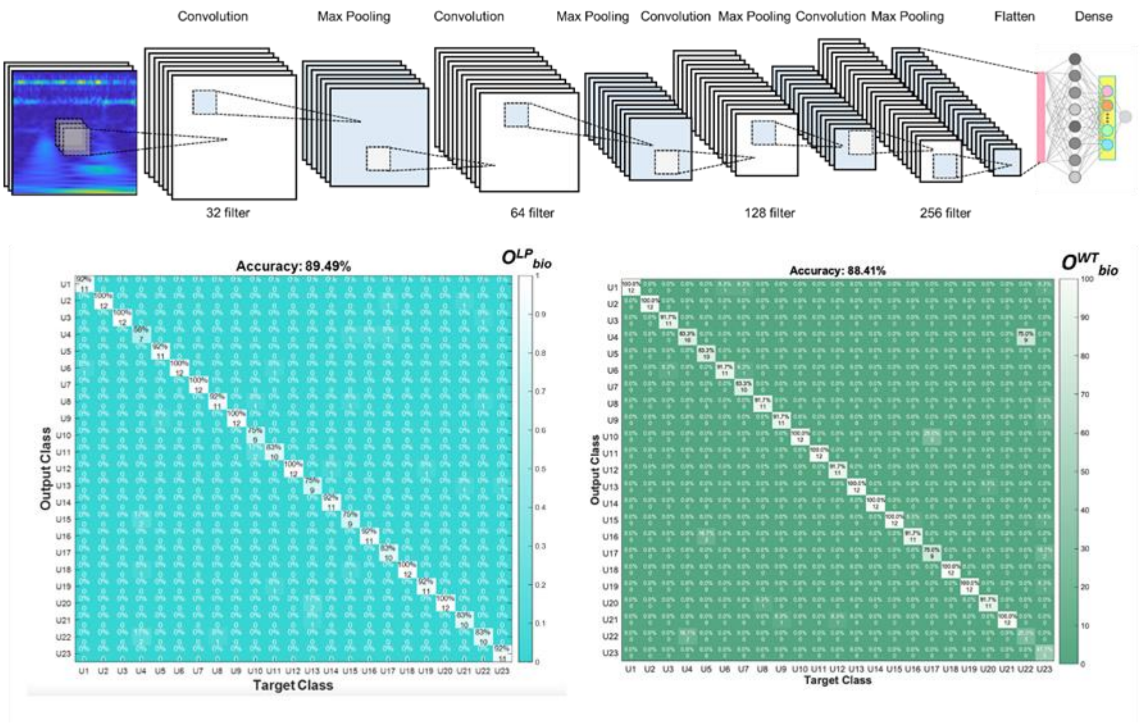

Fig. S32. Use CNN for user identification using the converted 2D map obtained from wavelet transform.

The accuracy of recognition using signals after wavelet transformed ($O^{WT}{}_{bio}$) is comparable to the accuracy of recognition using instantaneous time-varying signals ($O^{LP}{}_{bio}$).

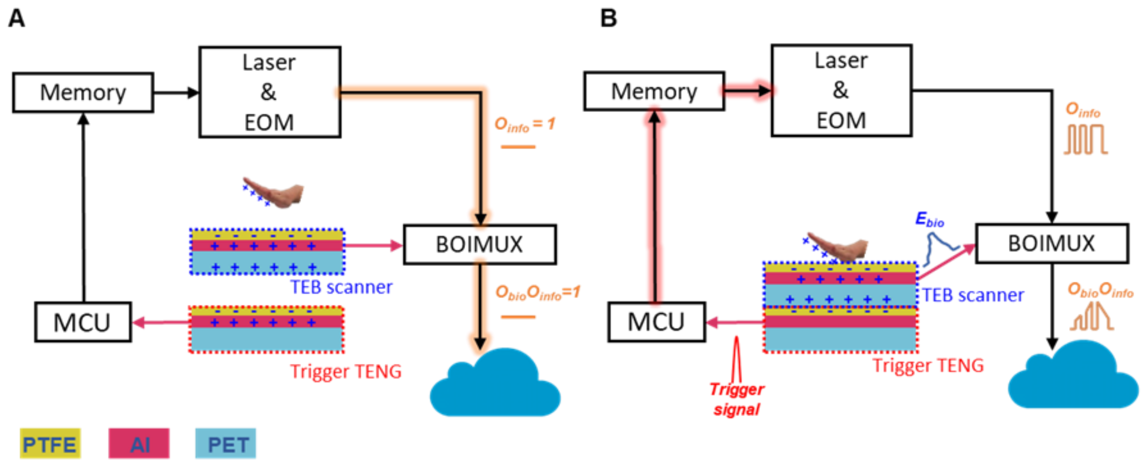

Fig. S33. Combination of the TEB scanner with a trigger TENG to ensure the simultaneous generation of digital information and biometric information.

(A) The optical information is a constant 'ONE' before palm interaction. **(B)** Simultaneous generation and multiplexing of digital information and biometric information upon palm interaction.

Movie Legends

Movie S1. Mechanism of file upload using biometrics-protected optical communication system

Movie S2. Real-life demonstration of file upload using biometrics-protected optical communication system

Movie S3. Mechanism of file request using biometrics-protected optical communication system

Movie S4. Real-life demonstration of file request using biometrics-protected optical communication system

Movie S5. Real-life demonstration of biometrics-protected smart home control in VR space
